# Inhibition of hypoxia inducible factors combined with all-*trans* retinoic acid treatment enhances glial transdifferentiation of neuroblastoma cells

**DOI:** 10.1038/srep11158

**Published:** 2015-06-09

**Authors:** Flora Cimmino, Lucia Pezone, Marianna Avitabile, Giovanni Acierno, Immacolata Andolfo, Mario Capasso, Achille Iolascon

**Affiliations:** 1 Dipartimento di Medicina Molecolare e Biotecnologie Mediche, Università degli Studi di Napoli “Federico II”, Naples, Italy; 2 CEINGE Biotecnologie Avanzate, Naples, Italy; 3 Scuola di Medicina e Chirurgia, Università degli Studi di Verona, Verona, Italy

## Abstract

Neuroblastoma (NBL) is a heterogeneous tumor characterized by a wide range of clinical manifestations. A high tumor cell differentiation grade correlates to a favorable stage and positive outcome. Expression of the hypoxia inducible factors HIF1-α (*HIF1A* gene) and HIF2-α (*EPAS1* gene) and/or hypoxia-regulated pathways has been shown to promote the undifferentiated phenotype of NBL cells. Our hypothesis is that *HIF1A* and *EPAS1* expression represent one of the mechanisms responsible for the lack of responsiveness of NBL to differentiation therapy. Clinically, high levels of *HIF1A* and *EPAS1* expression were associated with inferior survival in two NBL microarray datasets, and patient subgroups with lower expression of *HIF1A* and *EPAS1* showed significant enrichment of pathways related to neuronal differentiation. In NBL cell lines, the combination of all*-trans* retinoic acid (ATRA) with *HIF1A* or *EPAS1* silencing led to an acquired glial-cell phenotype and enhanced expression of glial-cell differentiation markers. Furthermore, *HIF1A* or *EPAS1* silencing might promote cell senescence independent of ATRA treatment. Taken together, our data suggest that HIF inhibition coupled with ATRA treatment promotes differentiation into a more benign phenotype and cell senescence *in vitro*. These findings open the way for additional lines of attack in the treatment of NBL minimal residue disease.

Neuroblastoma (NBL) is manifested in childhood as an extracranial solid tumor of the sympathetic nervous system, and it accounts for 15% of pediatric cancer deaths. NBL comprises cases with divergent outcomes that range from spontaneous differentiation/ regression to a benign tumor phenotype, to metastatic forms with poor prognosis^1^,^2^. The morphologically undifferentiated and the differentiated forms of NBL show different behaviors, where the undifferentiated form is mostly classified as aggressive tumors. In particular, the current International Neuroblastoma Pathology Classification guidelines identify a NBL subset with favorable prognosis using quantification of Schwannian stroma^3^,^4^.

NBL therapy and its intensity are based on risk stratification that takes into account both the clinical and biologic features that are known to be predictive of relapse. High-risk patients have tumors with unfavorable histological features, N-MYC amplification, and patient age at diagnosis <18 months^5^,^6^,^7^. Treatment of NBL includes radiation therapy and high-dose chemotherapy, followed by hematopoietic stem-cell transplantation. After recovery from myeloablative chemotherapy and stem-cell rescue, these patients are treated with the neuronal differentiating agent of oral isotretinoin (13-*cis* retinoic acid) for 6 months, to avoid potential minimal residual disease. Immunotherapy with antibodies developed to target GD2 is also given as part of the differentiation therapy regimen^8^,^9^. Despite recent improvements in survival in randomized trials, the patient outcome remains poor. Indeed, <50% of patients with high-risk NBL have a 5-year survival rate, contrary to the >90% 5-year survival rates for patients with low-risk NBL^6^,^7^. Improved knowledge of the neuronal differentiation pathways and the mechanisms of resistance might provide new and attractive targets for the development of new therapies that avoid tumor recurrence^10^,^11^.

Low oxygen tension in poorly vascularized areas has been associated with poor patient prognosis in solid tumors^12^,^13^. Adaptation of tumor cells to growth under hypoxic conditions has been primarily attributed to the accumulation of the hypoxia-inducible transcription factors HIF-1α (expressed by the *HIF1A* gene) and HIF-2α (expressed by the *EPAS1* gene). Increasingly, in a number of cancers, evidence has correlated HIF-1α overexpression under normoxia with poor prognosis, as an independent prognostic factor for poor chemotherapeutic response and shortened patient survival time. Factors such as nitric oxide^14^ and the cytokines interleukin-1beta (IL-1B) and tumor necrosis factor α (TNF-α)^15^, and trophic stimuli such as serum and the insulin-like growth factors^16^, might modulate HIF-1α up-regulation under normoxic conditions. Genetic alterations like overexpression of the *v-src* oncogene^17^ or inactivation of the tumor suppressor genes for p53, pVHL^18^ and PTEN^19^ might also enhance HIF expression and transcriptional activity. More importantly, up-regulation of HIF-1α levels in NBL tumors appears to be a significant mechanism for resistance to anti-angiogenic therapies, and suppression of HIF-1α levels with low-dose topotecan has been shown to potentiate the effects of anti-angiogenic drugs *in vivo*^20^,^21^.

In NBL cell lines, low oxygen tension might promote reductions in neuronal and neuroendocrine gene expression markers and the acquisition of an immature stem-like phenotype^22^,^23^. The correlation between hypoxia and the grade of the differentiation status suggests HIF and/or HIF-regulated pathways as one of the mechanisms behind the lack of cell responsiveness to differentiation therapy^24^. Therefore, inhibition of the HIFs might provide more effective methods to enhance the NBL cell propensity to differentiate.

Several studies *in vitro* have shown that in human NBL cell lines, the use of differentiating agents like all*-trans* retinoic acid (ATRA) and 13-*cis* retinoic acid can cause arrest of cell growth, and can also cause neuronal differentiation^25^,^26^,^27^. The aim of our study was to determine whether the combination of *HIF1A* or *EPAS1* silencing with ATRA treatment can provide major benefits over the use of the single agents. Our data show that ATRA alone induces neurite outgrowth and up-regulation of neural markers in RA-responsive NBL cells, whereas the combination of ATRA with *HIF1A* or *EPAS1* silencing drives the transdifferentiation of neuronal cells into Schwann-type cells, with cell senescence under long-term treatment. These effects might have great clinical impact for the treatment of minimal residue disease of patients with high-risk NBL, who are resistant to neuronal differentiation therapies. Overall, a full understanding of the mechanisms behind this transdifferentiation process should open up new opportunities for the development of novel therapies in the treatment of patients with NBL.

## Results

### Association of *HIF1A* and *EPAS1* expression with clinical outcomes in patients with NBL

In NBL cell lines, hypoxia-regulated pathways and/or HIF expression have been shown to promote an undifferentiated phenotype, either through dedifferentiation or through inhibition of differentiation. We speculated that *HIF1A* and *EPAS1* overexpression in patients with high-risk NBL will contribute to differentiation therapy resistance and to tumor cell aggressiveness.

**Figure 1:**
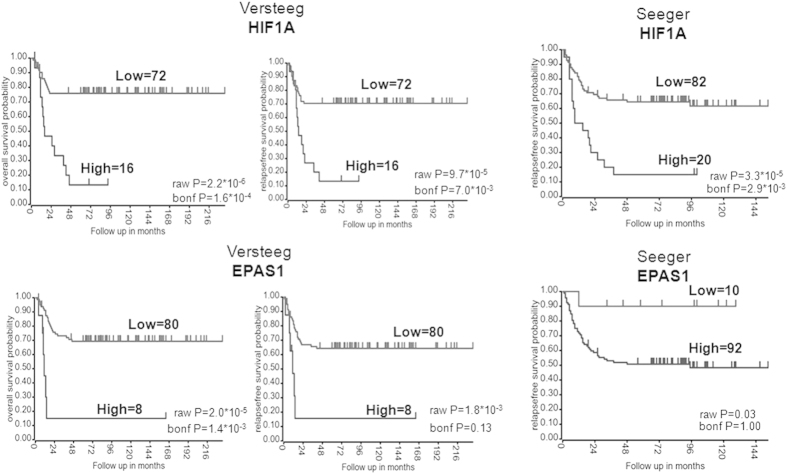
HIF1A and EPAS1 gene expression is associated with poor survival in patients with NBL. Kaplan-Maier analysis with patients grouped by the optimal cut-off (calculated using the R2 web tool) of expression of HIF1A and EPAS1 for overall survival and relapse-free survival rates, in 88 patients with NBL (Versteeg dataset) and 102 International Neuroblastoma Staging System stage 4 patients with MYCN not amplified (Seeger dataset). The overall survival data of the Seeger dataset were not available. The “raw P” indicates the uncorrected p-value, whereas the “bonf P” indicates the p-value corrected for multiple tests according the Bonferroni method.

We first evaluated the association of *HIF1A* and *EPAS1* expression with clinical outcomes in NBL patients using two datasets that are deposited in the R2 microarray web tool: the Seeger dataset that included 102 patients; and the Versteeg dataset that included 88 patients. The Seeger dataset includes patients with high-risk NBL (i.e., stage 4 disease), whereas the Versteeg dataset includes patients with different stages and ages at diagnosis. As shown in Fig. 1, high mRNA levels of *HIF1A* were significantly associated with lower overall survival and relapse-free survival in both sets of patients, whereas high expression levels of *EPAS1* showed significant association with lower overall survival and a trend toward an association with lower relapse-free survival, although this did not reach statistical significance. Furthermore, in the Versteeg dataset, high mRNA levels of *HIF1A* and *EPAS1* were still significantly associated with lower overall survival and relapse-free survival in the sub-set of patients with advanced-stage tumor (i.e., stage 4) (Supplementary Data, Fig. S1).

**Figure 2:**
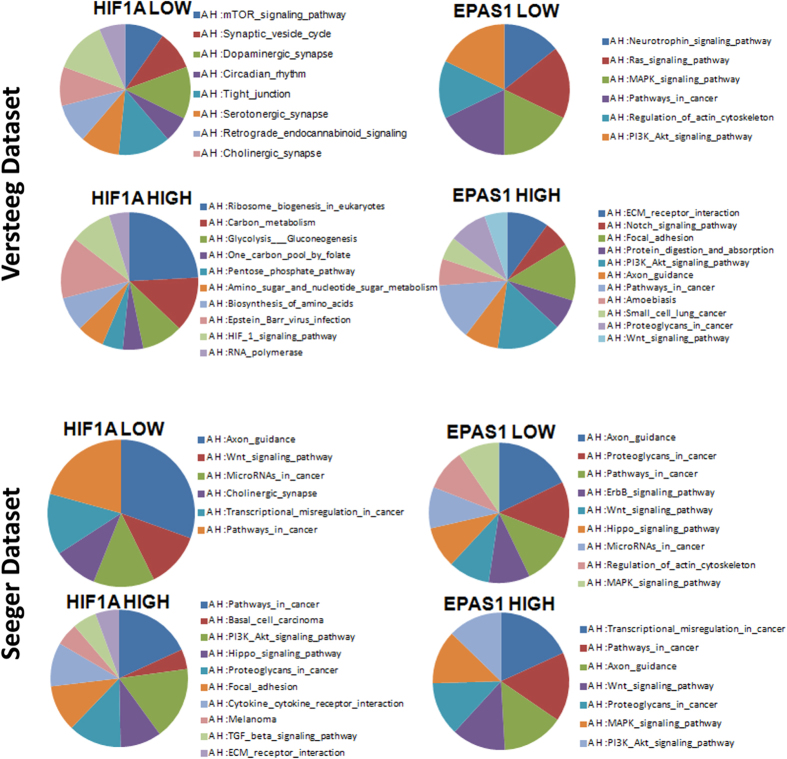
Pathway analysis of the genes involved in development that were differentially expressed between patients with ‘High’ and ‘Low’ levels of HIF1A and EPAS1 expression. Results of the pathway analysis for the genes involved in development in patients with ‘High’ and ‘Low’ mRNA levels of *HIF1A* and *EPAS1* (P ≤ 0.05).

In both of these datasets, we identified two patient subgroups with different expression levels of *HIF1A* and *EPAS1*, in terms of their ‘High’ and ‘Low’ expression levels. We investigated whether these genes that are differentially expressed between these two subgroups can influence the neuronal differentiation pathways. We filtered the genes included in the category “Development” (Supplementary Data, Tables S1, S2) and we performed gene ontology analysis. As shown in Fig. 2, the neuronal differentiation pathways were more represented in the patient group for ‘Low’ *HIF1A* or *EPAS1* expression than for ‘High’ *HIF1A* or *EPAS1* expression (P ≤ 0.05). We also observed that MAPK PI3K-AKT signaling pathways were represented in the ‘High’ and ‘Low’ *EPAS1* expression subgroups in both datasets^28^. Interestingly, the *IL-1B*, *IKBKB*, *RELA* and *NFKB1* genes were more expressed in the ‘High’ (*HIF1A* and *EPAS1*) expression subgroups in the Versteeg dataset, while the same factors were overexpressed only in the ‘High’ *HIF1A* expression subgroup in the Seeger dataset^15^ (Supplementary Data, Fig. S2).

### *HIF1A* and *EPAS1* silencing in NBL cells

We then selected three NBL cell lines: SHSY5Y, SKNBE2c and SKNAS cells. The SHSY5Y and SKNBE2c cell lines have biochemical features of neuronal cells, and these are believed to represent embryonic precursors of sympathetic neurons. These cells differentiate toward a neuronal phenotype upon RA treatment. The SKNAS cells have the flat phenotype of glial cells, and they do not differentiate^25^,^26^.

**Figure 3:**
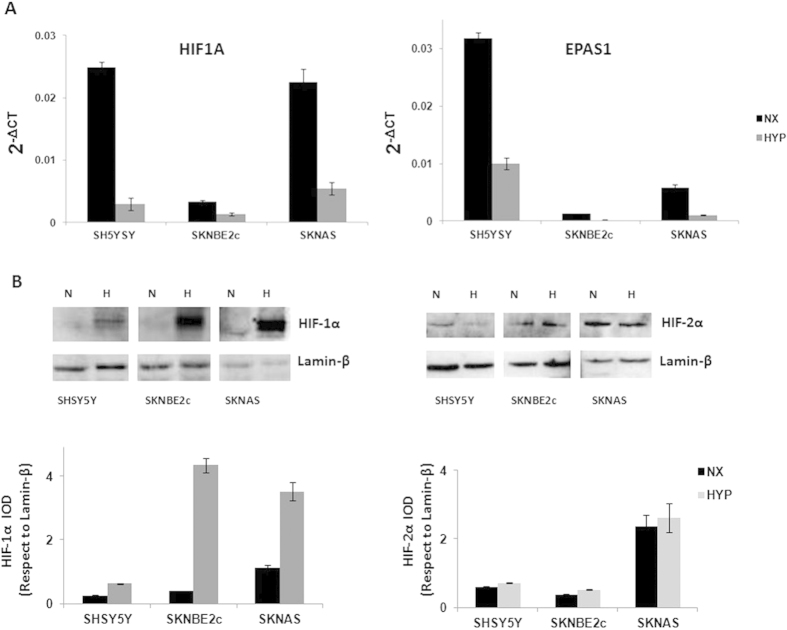
*HIF1A* and *EPAS1* and HIF-1α and HIF-2α protein expression under normoxia and hypoxia conditions. The three cell lines (SHSY5Y, SKNBE2c, SKNAS) were grown in normoxia (NX) and hypoxia (HYP) (1% oxygen for 6 h). The relative mRNA expression of *HIF1A* and *EPAS1* normalized to β-actin expression as determined (2^−ΔCT^) from the RT-PCR analysis (**A**). Western blotting of nuclear extract from the cells grown under normoxia (**N**) and hypoxia (**H**) conditions shows HIF-1α and HIF-2α changes under hypoxia. The bands were quantified by densitometry. The bar graphs shows the integral optical density (IOD) for each band, normalized with respect to lamin-β expression (**B**). Data are means of three experiments.

These three cell lines showed different basal levels of *HIF1A* and *EPAS1* expression, as shown by their 2^−ΔCT^ values, which represent their relative gene expression. The SHSY5Y and SKNAS cells had higher levels of *HIF1A* expression than the SKNBE2c cells. *EPAS1* expression was higher in the SHSY5Y cells with respect to the SKNBE2c and SKNAS cells. The SKNBE2c cells showed the lowest levels of both *HIF1A* and *EPAS1* expression, with respect to the SHSY5Y and SKNAS cells (Fig. 3A). Here, the fold-differences in the levels of expression of *HIF1A* and *EPAS1* among these three cell lines were determined using the mean differences in the ∆C_T_ between each of the SHSY5Y and SKNAS cells and the SKNBE2c cells (i.e., as the internal control), as shown in the Supplementary Data (Fig. S3).

In the same cells grown under hypoxic conditions, these *HIF1A* and *EPAS1* relative mRNA levels (2^−ΔCT^) were decreased with respect to those in the cells grown under normoxic conditions (Fig. 3A, HYP, NX, respectively). The mean fold-changes between the hypoxic and normoxic conditions were determined according to the mean differences in the ∆C_T_ between *HIF1A* and *EPAS1* under these conditions (with normoxic as the internal control; Supplementary Data, Fig. S3). As shown by the Western blotting in Fig. 3B, the expression levels of the HIF-1α and HIF-2α proteins (as determined by the integral optical densities [IODs], and normalized with respect to lamin-β expression) were stabilized in these NBL cells grown under hypoxic conditions. Expression of the HIF-1α protein was more stabilized than for the HIF-2α protein. These data suggest that the *HIF1A* and *EPAS1* mRNA levels decreased probably because the increases in the HIF-1α and HIF-2α protein levels would result in negative feedback on their mRNA production.

**Figure 4:**
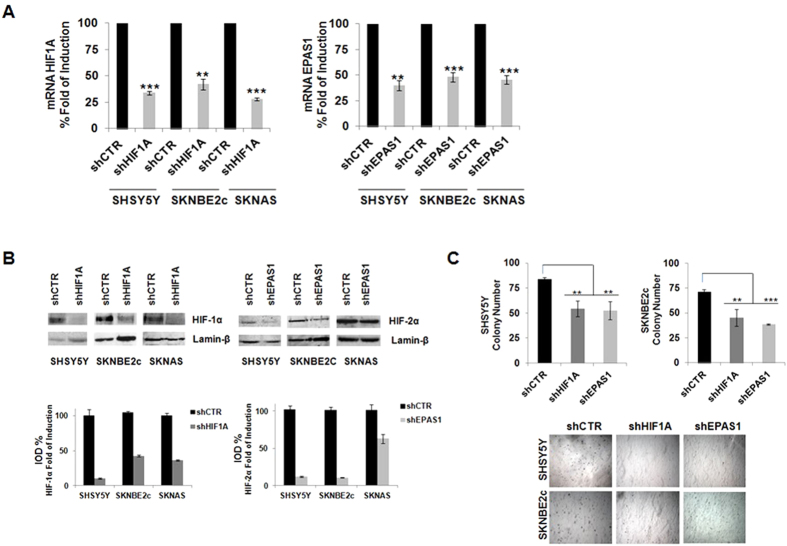
*HIF1A* and *EPAS1* silencing in NBL cells and the effects on cell growth in the colony formation assay. (**A**) Efficiency of gene silencing mediated by lentiviral delivery of hairpin RNAs directed against *HIF1A* and *EPAS1* (shHIF1A, shEPAS1, respectively) in the three NBL cell lines (SHSY5Y, SKNBE2c, SKNAS) was assessed using RT-PCR. Data are means of three experiments and are represented as percentages with respect to the NBL shCTR cells, which were infected by lentivirus-mediated delivery of non-silencing hairpin RNA. (**B**) Western blotting of nuclear extracts from *HIF1A* and *EPAS1* silenced cells showing the decreases in the HIF-1α and HIF-2α proteins. The bands were quantified by densitometry. The bar graphs show the integral optical density (IOD) for each band, normalized with respect to lamin-β expression. (**C**) *HIF1A* and *EPAS1* silencing in SHSY5Y and SKNBE2c cells affects cell growth in the soft agar colony formation assay. The graft bars show decreased colony numbers in the NBL shHIF1A/shEPAS1 cells compared to the NBL shCTR cells. The SKNAS cells are not shown (** P ≤ 0.01; *** P ≤ 0.001).

The *HIF1A* and *EPAS1* silencing in these NBL cell lines under normoxic conditions was mediated using lentiviral delivery of short hairpin (sh)RNAs directed against *HIF1A* and *EPAS1* (i.e., shHIF1A and shEPAS1, respectively). A non-silencing shRNA was delivered using the lentivirus in the control cells (shCTR). The efficiency of silencing was determined by RNA expression (RT-PCR), as shown in Fig. 4A, where the gene expression is given as percentages relative to the shCTR cells (at 100%). In the gene silencing here, *HIF1A* mRNA expression was significantly decreased in the SHSY5Y (by 33.8%  ± 1.9%), SKNBE2c (by 42% ± 5.1%) and SKNAS (by 28% ± 1.3%) shHIF1A cells. *EPAS1* mRNA expression was also significantly decreased in the SHSY5Y (40% ± 4.8%), SKNBE2c (48% ± 12.0%) and SKNAS (46% ± 4.1%) shEPAS1 cells. These decreases in *HIF1A* and *EPAS1* mRNA were enough to influence the transcription of the target genes downstream of HIF, as shown in the Supplementary Data (Fig. S4).

The efficiency of the silencing was also determined by Western blotting in these three cell lines. As shown in Fig. 4B, there were decreases in the HIF-1α and HIF-2α protein levels upon *HIF1A* and *EPAS1* mRNA silencing, respectively. Moreover, these mRNA decreases were sufficient to reduce the oncogenic potential of these NBL cells, as shown by soft agar assays. Fig. 4C shows that there were decreases in the colony formation numbers in the SHSY5Y shHIF1A (54 ± 7.7 colonies) and shEPAS1 (52.5 ± 9.2 colonies) cells compared to the SHSY5Y shCTR cells (84 ± 4 colonies). Similarly for the SKNBE2c shHIF1A (45 ± 8.4 colonies) and shEPAS1 (38.5 ± 0.7 colonies) cells compared to the SKNBE2c shCTR cells (71 ± 2.8 colonies). The SKNAS cells showed weaker growth than the SHSY5Y and SKNBE2c cells^29^, and this depletion of the *HIF1A*/*EPAS1* genes did not change their growth (data not shown).

### Combination of ATRA with *HIF1A* or *EPAS1* silencing enhances the Schwann cell-like phenotype

Our initial goal was to determine the effects of *HIF1A* and *EPAS1* silencing on cell differentiation induced by ATRA in these NBL cells. SHSY5Y and SKNBE2c cells are RA responsive and SKNAS cells are RA unresponsive. These cell lines were silenced for *HIF1A* or *EPAS1* expression and then treated for 6 days with 5 μM and 10 μM ATRA. As reported in the literature, these ATRA concentrations can induce differentiation in diverse NBL cells without any toxicity resulting from such prolonged treatments^26^,^30^. It is known that ATRA treatment increases the expression of HIF-1α in acute promyelocitic leukemia and in other cell types^31^, and more remarkably, that HIF-1α inhibition cooperates with ATRA in the reduction of APL disease^32^. Here, we investigated the levels of the HIF-1α and HIF-2α proteins in these ATRA-treated NBL cells. Our data showed that ATRA treatment alone induced increases in the HIF-1α protein levels, as expected, and that *HIF1A* silencing combined with ATRA treatment prevented this ATRA-induced HIF-1α increase (Supplementary Data, Fig. S5). No increases in the HIF-2α protein were observed here. According to these data, inhibition of HIF in ATRA-treated tumors might synergize with ATRA, and so improve the success of this therapy.

**Figure 5:**
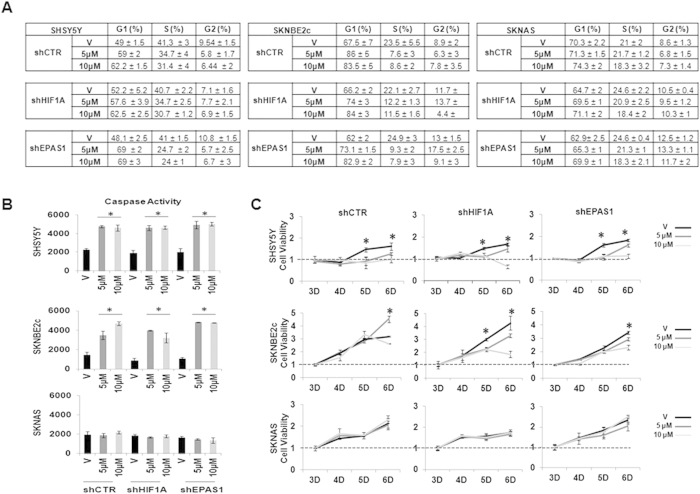
ATRA treatment: cell cycle and caspase activity. (**A**) Cells silenced for *HIF1A* or *EPAS1* expression were treated for 6 days with 5 μM or 10 μM ATRA and processed for flow cytometry using propidium iodide. The proportion of cells at each stage of the cell cycle were calculated. (**B**) Caspase-3 activity was assessed in the same cells (V, vehicle; 5 μM and 10 μM ATRA), and the data were normalized with respect to the negative control. (**C**) Cell viability was measured using the MTT assay, after 3, 4, 5, and 6 days (**D**) of ATRA treatment. Significances between the vehicle and the ATRA-treated cells are shown. The data are means of two experiments (* P < 0.05).

ATRA can induce differentiation in numerous NBL cell lines, which forces the cells out of the cell cycle. We examined the proportions of SHSY5Y, SKNBE2c and SKNAS cells at each stage of the cell cycle, through their caspase activities and their cell proliferation upon ATRA treatment and *HIF1A* or *EPAS1* silencing. Flow cytometry revealed that in the SHSY5Y shCTR, shHIF1A, and shEPAS1 cells and in the SKNBE2c shCTR, shHIF1A, and shEPAS1 cells, ATRA generated a modest accumulation of cells in G0/G1, with depletion of the cells in the S-phase and the G2 phase (Fig. 5A; Supplementary Data, Fig. S6). This depletion in the S-phase in response to ATRA treatment also showed increased caspase activities and reduced cell proliferation rates (Fig. 5B,C). Interestingly, the caspase activities in the SHSY5Y and SKNBE2c cells were similarly increased with ATRA treatment for the SHSY5Y and SKNBE2c shCTR, shHIF1A and shEPAS1 cells, which suggested that this co-treatment does not promote any increase in apoptosis. The absence of G1 accumulation and any increase in caspase activity, as well as the unvaried rate of proliferation in the treated SKNAS shCTR, shHIF1A, shEPAS1 cells indicated the failure of ATRA-induced cell differentiation in these cells.

**Figure 6:**
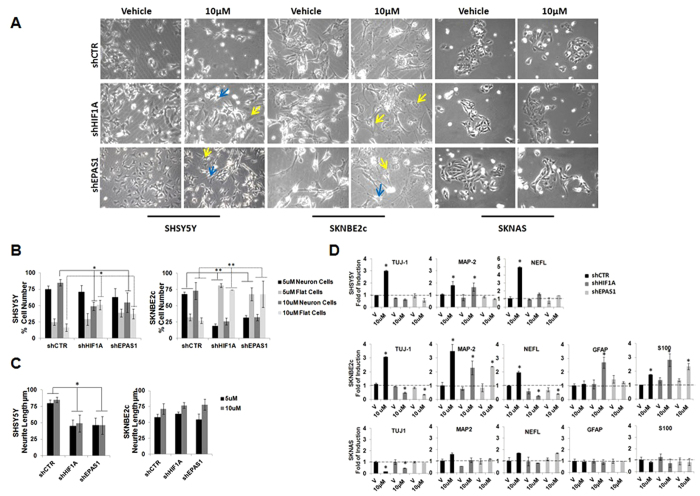
ATRA treatment combined with *HIF1A* or *EPAS1* silencing enhances the glial phenotype. (**A, B**) SHSY5Y, SKNBE2c, and SKNAS cells were silenced for *HIF1A* or *EPAS1* expression (shHIF1A, shEPAS1, respectively) and treated with 5 μM (not shown) and 10 μM ATRA, or only with vehicle (**V**), for 6 days. Phenotypic changes were analyzed by counting the cells that showed the neuronal and flat-cell phenotypes, in six different fields for each experimental point. The data are expressed as percentages. Blue arrows, examples of neuronal cells; yellow arrows, examples of flat cells. (**C**) The mean neurite lengths of the counted cells (μm). (**D**) Gene expression analysis of TUJ-1, NEFL, GFAP and S100 performed using RT-PCR in the three NBL cell lines. Data are means of three experiments (* P ≤0.05; ** P ≤ 0.01).

To estimate the neuronal differentiation under these treatments, we carried out a phenotype analysis, where the number of neuronal and flat cells were counted^33^, and these data are expressed as percentages (Fig. 6A). In the SHSY5Y shCTR cells, with the 5 μM and 10 μM ATRA treatments, there were more neuronal cells (75% ± 5%; 85% ± 5%; respectively) than flat cells (25% ± 5%; 16.6% ± 5.5%; respectively) (Fig. 6B). In contrast, upon 10 μM ATRA treatment in the SHSY5Y shHIF1 A and shEPAS1 cells, there were significant increases in the numbers of flat cells that were paralleled by decreases in the neuronal cells, compared to the SHSY5Y shCTR cells (Fig. 6B; P ≤ 0.05). In particular, in the SHSY5Y shHIF1 A cells, there were 49% ± 6.3% neuronal cells and 51% ± 7.0% flat cells, and in the SHSY5Y shEPAS1 cells there were 55% ± 1.5% neuronal cells and 37% ± 7.5% flat cells (Fig. 6B). These decreases in the neuronal-like cells at 5 μM and 10 μM ATRA treatment were accompanied by shorter mean neurite lengths in the SHSY5Y shHIF1A cells (38 ± 0.4 μm; 49 ± 13.0 μm) and the SHSY5Y shEPAS1 cells (44 ± 3.0 μm; 48 ± 10.8 μm), compared to the SHSY5Y shCTR cells (65 ± 16.0 μm; 63 ± 17.3 μm), (P ≤ 0.05) (Fig. 6C). In the SKNBE2c shCTR cells, there were increased numbers of neuronal cells with 5 μM ATRA (68% ± 3.8%) and 10 μM ATRA (73% ± 13.0%) treatment, with respect to the flat cells with 5 μM ATRA (32% ±5.4%) and 10 μM ATRA (27% ± 4.3%) treatment. In the SKNBE2c shHIF1A and shEPAS1 cells, instead, there were significantly increased numbers of flat cells with respect to a decrease in the number of neuronal cells, when compared to the SKNBE2c shCTR cells (P ≤ 0.005). In particular, for the SKNBE2c shHIF1A cells, upon 5 μM ATRA treatment there were 19% ± 3.7% neuronal cells and 81% ± 2.7% flat cells, and upon 10 μM ATRA treatment there were 26% ± 5.3% neuronal cells and 73% ± 0.1% flat cells. For the SKNBE2c shEPAS1 cells, upon 5 μM ATRA treatment there were 33%  ± 3.2% neuronal cells and 68% ± 10.0% flat cells, and upon 10 μM ATRA treatment there were 32% ± 5.0% neuronal cells and 68% ± 20.0% flat cells. The neuronal cell population did not show substantial differences in mean neurite length (Fig. 6B,C). The SKNAS shCTR, shHIF1A, and shEPAS1 cells did not show an obvious phenotypic responses to ATRA (Fig. 6A).

### Combination of ATRA with *HIF1A* or *EPAS1* silencing affects neuronal marker expression in NBL cells

We treated these NBL shCTR, shHIF1A and shEPAS1 cells for 6 days with 10 μM ATRA. To determine the neuronal differentiation, we investigated multiple factors that are known to be involved in axon guidance: beta-III-tubulin (TUJ-1) and microtubule-associated protein 2 (MAP-2), which are involved in microtubule assembly; neuronal light intermediate filament (NEFL), which is expressed during neuronal differentiation; glial fibrillary acidic protein (GFAP), which is expressed by astrocytes, and S100, which is expressed in cells derived from the neural crest (Schwann cells, melanocytes), both of which are implicated in the dynamics of cytoskeleton constituents.

As determined by RT-PCR, after this 10 μM ATRA treatment, in the SHSY5Y shCTR cells there was increased expression of the neuronal markers that was not seen in the SHSY5Y shHIF1A and shEPAS1 cells (except for MAP-2, the expression of which increased in the treated SHSY5Y shHIF1A cells) (Fig. 6D). Furthermore, all of these three SHSY5Y cells did not express glial markers at that time of treatment (data not shown). In the SKNBE2c shCTR cells, there were increases in neuronal marker expression, and in the SKNBE2c shHIF1A and shEPAS1 cells there were increases in glial marker expression. Although *HIF1A* or *EPAS1* silencing appeared to enhance the ATRA-induced glial marker expression, the glial markers were more highly expressed in the SKNBE2c shHIF1A cells than the SKNBE2c shEPAS1 cells. There were no differences in the expression of the neuronal and glial markers between the SKNAS shCTR, shHIF1A, and shEPAS1 cells (except for TUJ-1, the expression of which decreased in the ATRA-treated SKNAS shCTR cells compared to the ATRA-treated SKNAS shHIF1A and shEPAS1 cells).

**Figure 7:**
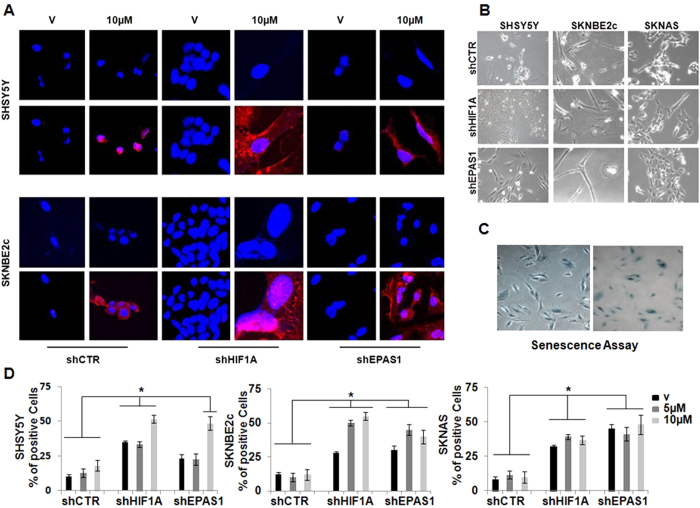
Long-term combination treatment of the NBL cell lines. (**A**) After 25 days of ATRA treatment, *HIF1A* or *EPAS1* silenced and ATRA-treated SHSY5Y and SKNBE2c cells were immunostained with an antibody against GFAP. The settings of the confocal microscope were strictly maintained throughout the whole study. The panels are representative of three independent experiments. (**B**) SHSY5Y and SKNBE2c shCTR cells show thread-like structures that indicate neuronal differentiation; whereas the SHSY5Y and SKNBE2c shHIF1A cells grow similar to ganglion structures, and the SHSY5Y and SKNBE2c shEPAS1 cells show a mixed population of flat and neuronal cells. The SKNAS cells did not show any morphological change. (**C,D**) After 40 days of treatment, the cells showed morphological evidence of senescence, which included the expression of senescence-associated-β-galactosidase (SA-β-Gal). Quantification was performed for the percentages of enlarged cells that showed SA-β-Gal expression. Data are means of three experiments (*P ≤ 0.05).

To determine whether this co-treatment might be more long-lived, we treated the cells with 10 μM ATRA for 25 days, and then assessed their differentiation status (Fig. 7A). Upon this extended 10 μM ATRA treatment, GFAP immunostaining was observed in the SHSY5Y and SKNBE2c shHIF1A and shEPAS1 cells, but not in vehicle-treated cells (Fig. 7A,V). In the ATRA-treated SHSY5Y and SKNBE2c shCTR cells, GFAP expression was very weak in the cytoplasm. The immunostaining of the glial marker GFAP in both of these cell lines supports the phenotypic changes in these SHSY5Y and SKNBE2c shHIF1A and shEPAS1 cells. As shown in Fig. 7B, the SHSY5Y shCTR cells preserved a neuronal-like phenotype, whereas the SHSY5Y shHIF1A and shEPAS1 cells gained a highly fusiform phenotype and formed pseudoganglia, which are typical of primary neurons. In the SKNBE2c shCTR cells, there was a mixed population of thread-like and flat cells, while the SKNBE2c shHIF1A and shEPAS1 cells showed glial phenotypes.

### Combination of ATRA with *HIF1A* or *EPAS1* silencing results in senescence of NBL cells

We also treated these NBL shCTR, shHIF1A, and shEPAS1 cells with 5 μM and 10 μM ATRA for 40 days, and then analyzed them for their senescence state. These final cell populations were tested for senescence-associated β-galactosidase (Fig. 7, SA-β-Gal)^34^. We observed that the vehicle-treated NBL shHIF1A and shEPAS1 cells showed greater senescence compared to the vehicle-treated NBL shCTR cells (Fig. 7, V; except for the SHSY5Y shEPAS1 cells). This suggested that the decreased *HIF1A* and *EPAS1* expression in these NBL cells might make them more prone to go into senescence, independent of the ATRA treatment. Furthermore, the combination of the ATRA treatment with the *HIF1A* or *EPAS1* gene silencing showed greater senescence in the SHSY5Y (10 μM ATRA) and SKNBE2c (5 μM, 10 μM ATRA) cells, but not in the SKNAS cells (Fig. 7C,D).

## Discussion

Neuroblastoma is an aggressive childhood tumor that derives from the peripheral neural crest. In the last 25 years, the long-term survival for high-risk NBL has improved, to reach about 50%^6^,^7^. Currently, there are constant efforts for the development of new therapeutic strategies against metastatic NBL, with the intention being to definitively avoid late recurrence. The clinical use of retinoids at high doses after intensive chemoradiotherapy (with or without autologous bone marrow transplantation) significantly improves event-free survival for patients with high-risk NBL^8^,^9^,^35^,^36^. However, resistance to therapy that appears to be caused by the drug pharmacokinetics or by several biochemical factors^37^ highlights an emergent demand for novel combination strategies with retinoids to provide major therapeutic efficiency.

Hypoxia is a condition of low oxygen tension that can push NBL cells toward an immature, stem-cell-like phenotype. Low oxygen levels mediate post-transcriptional regulation of the HIF-1α and HIF-2α proteins, and although these are structurally similar proteins, this might induce the expression of different genes in terms of the cell adaptation. The correlation between hypoxia and differentiating status grade would suggest that HIF and/or HIF-regulated pathways are one of the mechanisms that underlie the unresponsiveness to differentiation therapy^22^,^23^,^24^. Overexpression of HIF-1α has been observed in a wide spectrum of solid tumors, even under normoxia conditions. The mechanisms that might regulate HIF-1α expression and ultimately lead to increased tumor growth and chemoradioresistance are different^38^,^39^. Otherwise, HIF-2α is more tissue-specific, and it promotes the growth of clear-cell renal cell carcinoma (ccRCC) and NBL cells, and it is involved in the regulation of stem cell maintenance^40^,^41^. A multitude of compounds have thus been developed to directly interfere with HIF-1α^42^,^43^,^44^. Therefore, there is increasing interest in the identification of the mechanisms of HIF-1α up-regulation in solid tumors, to guide the choice of HIF inhibitors (e.g., transcription- or translation-based) that will be best-suited for treatment^45^,^46^.

In the present study, we initially showed that *HIF1A* and *EPAS1* mRNA levels correlate with worse survival in high-risk patients with NBL, using two different NBL microarray gene-expression datasets. In both of these datasets, two patient subgroups were identified that showed ‘High’ and ‘Low’ *HIF1A* and *EPAS1* expression levels. As a result of our gene ontology analysis that was restricted to developmental genes that were differentially regulated between these two subgroups for both of the datasets, we observed that neuronal pathways were more represented in patients with ‘Low’ *HIF1A* or *EPAS1* expression, than in those with ‘High’ *HIF1A* or *EPAS1* expression. This finding suggests that low expression of *HIF1A* or *EPAS1* in tumors that are more differentiated might promote an efficient NBL cell response to differentiation therapy. According to the literature, *HIF* gene overexpression in NBL solid tumors might be determined by several factors, as well as a hypoxic microenvironment. In our analysis here, we observed that the MAPK PI3K-AKT pathways were more represented in the patient subgroups with different *EPAS1* expression, which suggests that these pathways might orchestrate the regulation of the *EPAS1* mRNA in these tumors^28^. Moreover, we addressed the hypothesis that IL-1B might up-regulate HIF-1α in patients with NBL via a pathway that is dependent on nuclear factor kappa B (NF*K*B)^15^. Also, we could not exclude that *HIF1A* and/or *EPAS1* expression in solid tumors is regulated by other factors, such as genetic/ epigenetic alterations, which were not determined in the present study.

In NBL cell lines, we observed that a low oxygen concentration stabilized HIF-1α and HIF-2α protein expression, whereas the *HIF1A* and *EPAS1* mRNA levels decreased. However, the increases in the HIF-2α protein levels during hypoxia were only modest in comparison to those for HIF-1α protein levels in the same cells. These protein increases in hypoxia might result in negative feedback on their mRNA production, thus suggesting that high *HIF1A* and *EPAS1* gene expression under normoxic growth conditions might be determined by other factors that are not directly linked to the low oxygen concentration.

Based on these findings, our aim was then to dissect out the role of *HIF1A* and *EPAS1* expression in NBL cell lines, regardless the microenvironment. Indeed, *HIF1A* and *EPAS1* mRNA expression in normoxia might reflect the results obtained in our *in-silico* analysis of NBL microarrays, and *HIF1A* and *EPAS1* silencing might represent a useful therapeutic approach for the treatment of solid tumors with high *HIF1A* and/or *EPAS1* mRNA levels. Moreover, hypoxia might generate O_2_-level-dependent reprogramming, the downstream effects of which will not be strictly connected to *HIF1A* and *EPAS1* expression^47^,^48^.

In the present study, we have defined a new combination treatment in NBL cell lines that drives glial differentiation and senescence responses. This treatment is based upon the use of *HIF1A* or *EPAS1* gene silencing to enhance ATRA-induced differentiation proprieties. We observed that this mechanism operates in certain NBL cells (i.e., the SHSY5Y and SKNBE2c cells) but not in cells that are resistant to retinoids (i.e., the SKNAS cells). Our findings show that when ATRA is used alone, it induces neurite outgrowth and up-regulation of neural markers, whereas the combination treatment of *HIF1A* or *EPAS1* gene silencing and ATRA treatment enhances the glial phenotype and promotes up-regulation of glial markers. More significantly, even with extended *HIF1A* or *EPAS1* silencing and ATRA co-treatment, these cells were driven into ganglion-like clusters, and thus resembled less aggressive ganglioneuroma cells.

Upon ATRA treatment, we observed that the *EPAS1*-silenced cells (i.e., NBL shEPAS1 cells) showed an intermediate phenotype between the unsilenced (i.e., the NBL shCTR cells) and the *HIF1A*-silenced cells (i.e., the NBL shHIF1A cells), which suggests that *EPAS1* is not the main player in determining neuronal-glial transdifferentiation. The individual *HIF1A* or *EPAS1* gene silencing was not sufficient to lead to subdifferentiation into Schwann cells, as also for ATRA used as a single agent, which acts independent of Schwann cell differentiation. The combination treatments of *HIF1A* or *EPAS1* silencing in the presence of ATRA exclusively pushed the NBL cells toward transdifferentiation of the neuronal cells into Schwann cells. These NBL cell lines are characterized by markers of embryonic peripheral nervous system development. Of note, these cells showed a continuum process in their differentiation between the neuronal and glial lineages, which resembled normal peripheral nervous system development^49^. Therefore, our findings suggest that *HIF1A* or *EPAS1* (or HIF-related pathways) sustain the activation of alternative pathways that can provide Schwann transdifferentiation in NBL cells upon retinoid treatment. The conversion of NBL cells into ganglionic and Schwann cells might have great clinical impact in differentiation therapeutic protocols. Indeed, ganglionic/ Schwann cells might induce the secretion of several substances that have antitumor properties^50^,^51^, and as a consequence this might improve the therapeutic success of such combination therapies.

Pro-senescence therapy has been argued as a promising therapeutic strategy for the treatment of cancers. In NBL, tumor relapse is the most significant barrier to effective therapy, and compounds such as the retinoid agents that induce initial neuronal differentiation might fail to prevent relapse. The proposed combination therapy can potentially help to reduce NBL relapse through two main effects: (i) differentiation into a more benign phenotype; and (ii) induction of senescence. RA can cause senescence in some NBL cells^52^,^53^, but it is currently not clear whether senescence represents a significant component of its clinical response. Evidence from the literature has correlated senescence and hypoxia; in particular, hypoxia can inhibit or prevent senescence in cells, even if the pathways that are altered remain unknown^54^. In our cell systems, we showed that silencing of *HIF1A* or *EPAS1* expression is enough to increase the number of senescent cells independent of the ATRA responsiveness, and that the combination of ATRA with *HIF1A* or *EPAS1* silencing enhances senescent cells in RA-responsive cells.

Taken together, our findings provide the first evidence that *HIF1A* or *EPAS1* inhibition combined with ATRA treatment can convert NBL cells into ganglionic and Schwann cells and might also generate a novel trigger for senescence in NBL RA-responsive cells. Indeed, these data shed new light in the mechanisms underlying the neuronal–Schwann cell transdifferentiation process, which might represent a model for the development of novel therapeutic strategies for patients with high-risk NBL^55^. In particular the introduction of HIF inhibitors into differentiation therapy protocols might offer therapeutic advantages for relapse prevention, which represents a significant barrier that needs to be surmounted in therapeutic approaches for patients with high-risk NBL.

## Materials and methods

### Microarray-KAPLAN SCAN

We used the R2 web tool (http://r2.amc.nl)^56^ to predict the association of *HIF1A* and *EPAS1* expression with survival of patients with NBL. In brief, for each gene, R2 calculates the optimal cut-off in the expression level to divide the patients into ‘good’ and ‘bad’ prognosis cohorts. Samples within a dataset are sorted according to the expression of the investigated genes, and are divided into two groups. All of the cut-off expression levels and their resulting groups are analyzed according to patient survival. For each cut-off level and grouping, the log-rank significance of the projected survival is calculated. The best P value and the corresponding cut-off are selected. The cut-off level is reported and was used to generate the Kaplan-Meier curves. These depict the log-rank significance (raw P) as well as the P value corrected for multiple testing (Bonferroni correction) of the cut-off levels for each gene. Kaplan scan analysis was performed to estimate the overall survival and relapse-free survival according to *HIF1A* and *EPAS1* in the two microarray datasets: the Seeger dataset that included 102 International Neuroblastoma Staging System stage 4 patients without *MYCN* amplification; and the Versteeg dataset that included 88 patients with different clinical characteristics.

### Correlation of genes involved in development and *HIF1A* and *EPAS1* mRNA levels

Differential expression of genes involved in development was tested between the two groups of patients divided according to their median values of expression of *HIF1A* and *EPAS1*. This analysis was performed using the R2 web tool (http://r2.amc.nl) and the gene-expression data of the Versteeg and Seeger datasets. The list of genes for both of these datasets is shown in Supplementary Table S1 and Table S2. The coefficient of correlation (R-value) between the gene expression values and the two subgroups (‘High’ and ‘Low’ *HIF1A* or *EPAS1* espression) is also reported. The statistical differences in the gene expression values between the patient groups with ‘High’ and ‘Low’ *HIF1A* or *EPAS1* expression were evaluated by ANOVA tests implemented in the R2 web tool. The p-values were corrected for multiple testing according to the false discovery rate^57^. The significant threshold was established at a false discovery rate of 30%. Kegg pathway analysis was independently performed on the two lists (Supplementary Tables S1, S2) of the significant genes obtained from the two datasets (i.e., Versteeg, Seeger).

### Cell culture

The human SHSY5Y, SKNBE2c, SKNAS and HEK293T cell lines were grown in Dulbecco’s modified Eagle’s medium supplemented with 10% heat-inactivated fetal bovine serum (Sigma), 1 mM L-glutamine, penicillin (100 U/ml) and streptomycin (100 μg/ml) (Invitrogen), at 37 °C, under 5% CO_2_ in a humidified atmosphere. The NBL cells were plated at 60% to 70% confluence and treated with 5 μM and 10 μM ATRA (Sigma) dissolved in dimethyl sulfoxide. During the *HIF1A* and *EPAS1* silencing and the ATRA treatments, the cells were grown under normoxic conditions. The cells under hypoxia were grown at 1% oxygen for 6 h.

### Production of lentiviral particles and infection of cell lines

To knock-down *HIF1A* and *EPAS1* expression, the pGIPZ lentiviral shRNAmir that targets human *HIF1A* and *EPAS1* were purchased from Open Biosystems (Thermo Fisher Scientific, Inc.). We used two different shRNAs for each gene. The shRNAs against *EPAS1* were: V2LHS113750 (RHS4430-98894439) and V2LHS-113750 (RHS4430-98851126). The shRNAs against *HIF1A* were: V2LHS_132152 (RHS4430-98513964) and V2LHS_236718 (RHS4430-98513880). A non-silencing pGIPZ lentiviral shRNAmir was used as the control (RHS4346).

HEK293T were transfected using 10 μg shRNA plasmid DNA, 30 μl Trans-Lentiviral Packaging Mix (OpenBiosystem), and 25 μl TrasFectin (Bio-Rad), in 10-mm plates. The supernatants (10 ml per condition) were harvested after 24 h, centrifuged at a low speed to remove cell debris, and filtered through 0.45-μm filters. *In-vitro* transduction and determination of the lentivector titre were performer as reported previously^58^. After 48 h of incubation, the transduced cells were examined microscopically for the presence of TurboGFP expression (70%–90%). To obtain 100% GFP-positive cells, puromycin was added into the medium for an additional 10 days. The reported data are representative of the experiments performed and confirmed using both lentiviral vectors for each gene.

### Fractionation of nuclear proteins and Western blotting

Cell pellets were resuspended in a hypotonic buffer (10 mM HEPES-K^+^, pH 7.5, 10 mM KCl, 1.5 mM MgCl_2_, 0.5 M dithiothreitol) in the presence of a protease inhibitors cocktail (Roche). The cells were lysed by addition of ice-cold 0.5% NP-40 for 10 min. The nuclei were pelleted at 1,000 x *g* for 2 min at 4 °C. The nuclear pellets were washed twice with hypotonic buffer, and then resuspended in nucleus lysis buffer (20 mM HEPES-K^+^, pH 7.9, 420 mM NaCl, 0.2 mM EDTA, 1.5 mM MgCl_2_, 0.5 M dithiothreitol, 25% glycerol) with protease inhibitors. The nuclei were incubated on ice for 30 min and vortexed periodically. The supernatants containing the nuclear proteins were collected by centrifugation at 16,000 x *g* for 5 min at 4 °C. The protein concentrations were determined by Bradford assays (Bio-Rad). Thirty micrograms of protein were loaded and separated using 8% polyacrylamide gels, and transferred onto polyvinylidene difluoride membranes (Bio-Rad). The membranes were blocked with 5% non-fat dried milk (Applichem) in phosphate-buffered saline (PBS) with 0.2% Tween (PBS-T) for 1 h, and then probed with anti-HIF-1α (610959; BD Biosciences) or anti-HIF-2α (ab8365; Abcam) antibodies. After a wash in PBS-T, the membranes were incubated with horseradish-peroxidase-conjugated anti-mouse secondary antibody (1:4,000 dilution; ImmunoReagent), and then the positive bands were visualized using the ECL kit SuperSignal West Pico Chemiluminescent Substrate (Pierce). A goat anti lamin-β antibody (1:100 dilution; sc-6216; SantaCruz) was used as the control for equal loading. The protein band images were acquired with a GelDoc 2000 system (Bio-Rad), and the densitometry measurements were performed using the Quantity One 4.5 tool (Bio-Rad).

### Colony formation assay in soft agar

The colony formation assay was performed to analyze anchorage-independent cell growth. Two hundred thousand cells were plated in 0.35% agar on a bottom layer of 1% agar in the 35-mm dishes of 6-well plates (Corning). The plates were incubated at 37 °C for 4 weeks, and then stained with 0.01% crystal violet. Colonies with 20 cells or more were counted. The means and standard deviations were calculated from three independent experiments.

### Cell cycle distributions

Cells were seeded in cell culture 10-mm × 20-mm dishes (Corning) at a density of 1 × 10^6^ cells. After 8 h of serum starvation, the cells were treated with 5 μM and 10 μM ATRA for 24 h and 48 h. For the cell cycle analysis, 1 × 10^6^ cells were washed in PBS and resuspended in 200 μl propidium iodide (50 μg/ml in PBS; Sigma), plus 50 μl RNaseA solution (100 μg/ml in water; Sigma) and 0/004% NP40 in PBS. The cells were incubated at 37 °C for 3 h in the dark. The cell-cycle distribution was then analyzed using flow cytometry, by fluorescence-activated cell sorting analysis (BD FACS, Canto II, BD Biosciences). The means and standard deviations were calculated from two independent experiments.

### Caspase-3 activity assay

Caspase-3 activity was evaluated using Caspase Fluorescent (AFC) Substrate/ Inhibitor QuantiPak (ENZO Life Sciences), following the manufacturer protocol. Briefly, cell lysates (total protein, 100 μg) were added to reaction mixtures (final volume, 100 μl) containing fluorigenic substrate peptides that are specific for caspase 3 (DEVD-AFC). The reaction was performed at 37 °C for 1 h. Fluorescence was measured with a fluorescence microplate reader (Microplate Imaging System, Bio-Rad), at 530 nm.

### Cell viability assay

Cells were grown in the presence of ATRA for a total of 6 days. After 2 days, the cells were seeded as six replicates into 96-well plates at a density of 10^4^ cells per well. After 3, 4, 5, and 6 days of treatment, the metabolic activities of the samples were assessed as a surrogate marker for cell proliferation, using the 3-(4,5-dimethylthiazol-2-yl),5-diphenyltetrazolium bromide assay, according to the manufacture protocol (Promega).

### Quantification of neurite outgrowth

Neurite outgrowth was defined as neurite processes that were equal to or greater than two times the length of the cell body^54^. Neurites as single, dispersed cells were measured from the cell body to the furthest tip of the process using the LeicaApplicationSuite/AF software and a DMI4000B microscope (Leica Mycrosystem). The means and standard deviations of the neurite populations were calculated from three independent experiments.

### Real-time RT-PCR

The expression levels of seven genes were analyzed using real-time, quantitative PCR. Total RNA extraction using TRIzol LS Reagent (Invitrogen) and cDNA retrotranscription using the High Capacity cDNA Reverse Transcription Script (Applied Biosistem) was performed according to the manufacturer protocol. The cDNA samples were diluted to 20 ng/μl. Gene-specific primers were designed by using PRIMEREXPRESS software (Applied Biosystems), as: HIF1A Forward (F) CCCATAGGAAGCACTAGACAAAGT, Reverse (R) TGACCATATCACTATCCACATAAA; EPAS1 (F) GACCCAAGATGGCGACATG, (R) TGTCCTGTTAGCTCCACCTGTG; TUJ-1 (F) TATGAGGGAGATCGTGCACATC, (R) TGACTTCCCAGAACTTGGCC; MAP-2 (F) TCTCTTCTTCAGCGCACCGGCG, (R) GGGTAGTGGGTGTTGAGGTACC; NEFL (F) AAGCATAACCAGTGGCTACTCCCA, (R) TCCTTGGCAGCTTCTTCCTCTTCA; GFAP (F) GGTTGAGAGGGACAATCTGGC, (R) GCTTCCAGCCTCAGGTTGG; S100 (F) GCTGAAAGAGCTGCTGCAGA, (R) TTCATACACCTTGTCCACAGCAT; β-Actin (F) CGTGCTGCTGACCGAGG, (R) GAAGGTCTCAAACATGATCTGGGT. Real-time PCR was performed using SYBR Green PCR Master Mix (AppliedBiosystems). All real-time PCR reactions were performed using the 7900HT Fast Real-Time PCR System (Applied Biosystems). The experiments were carried out in triplicate for each data point. The housekeeping gene β-actin was used as the internal control. Relative gene expression was calculated using the 2^−ΔCT^ method^59^, where the ∆C_T_ was calculated using the differences in the mean C_T_ between the selected genes and the internal control (β-actin). The mean fold change of 2^−(average ∆∆CT)^ was determined using the mean difference in the ∆C_T_ between the gene of interest and the internal control^59^.

### Immunofluorescence

Cells were placed on the chambers of polystyrene vessel tissue culture treated glass slides (BD Falcon), fixed in 4% paraformaldehyde, permeated with 0.2% Triton X-100, and blocked with 1% bovine serum albumin. The anti-GFAP (SAB4100002, Sigma) primary antibody was incubated for 1 h to 3 h, and the AlexaFluor 546 donkey anti-mouse (A10036, Invitrogen) secondary antibody for 45 min. Nuclear staining was obtained using 4’,6-diamino-2-phenylindole (DAPI, Roche) (1:10,000 dilution in methanol).

Confocal microscopy images of cells were acquired using a point-scanning confocal microscope (Zeiss LSM 510 Meta; Zeiss, Germany) with a 40 × EC Plan-Neofluar oil-immersion objective. Digital images were acquired using the LSM 510 Meta software^60^. All of the instrumental parameters for the fluorescence detection and image analyses were held constant to allow cross-sample comparisons.

### Senescence analysis

Senescence of cells during differentiation experiments was analyzed using Senescence β-Galactosidase Staining kits (Cell Signaling Technology)^34^. Briefly, the cells were fixed in 2% glutaraldehyde/ 20% formaldehyde and then stained at 37 °C overnight with the X-gal staining solution. The blue cells were considered positive. The means and standard deviations were calculated from three independent experiments.

### Statistical analysis

The differences between the groups were analyzed using unpaired student’s t-test. Probability values <0.05 were considered to be statistically significant. * P ≤ 0.05, ** P ≤ 0.01, *** P ≤ 0.001.

## Additional Information

**How to cite this article**: Cimmino, F. *et al*. Inhibition of hypoxia inducible factors combined with all-*trans* retinoic acid treatment enhances glial transdifferentiation of neuroblastoma cells. *Sci. Rep*. **5**, 11158; doi: 10.1038/srep11158 (2015).

## Supplementary Material

Supplementary Information

## References

[b1] Diede S. J. Spontaneous regression of metastatic cancer: learning from neuroblastoma. Nat Rev Cancer. 14, 71–2 (2014). 24616911 10.1038/nrc3656

[b2] Maris J. M. Recent advances in neuroblastoma. N Engl J Med. 362, 2202–11 (2010). 20558371 10.1056/NEJMra0804577 PMC3306838

[b3] Tornóczky T., Semjén D., Shimada H. & Ambros I. M. Pathology of peripheral neuroblastic tumors: significance of prominent nucleoli in undifferentiated/poorly differentiated neuroblastoma. Pathol Oncol Res. 13, 269–75 (2007). 18158560 10.1007/BF02940304

[b4] Mohlin S. A., Wigerup C. & Påhlman S. Neuroblastoma aggressiveness in relation to sympathetic neuronal differentiation stage. Seminars in Cancer Biology 21, 276–282 (2011). 21945591 10.1016/j.semcancer.2011.09.002

[b5] Davidoff A. M. Neuroblastoma. Semin Pediatr Surg. 21, 2–14 (2012). 22248965 10.1053/j.sempedsurg.2011.10.009 PMC3261589

[b6] Maris J. M., Hogarty M. D., Bagatell R. & Cohn S. L. Neuroblastoma. Lancet. 23, 2106–20 (2007) 10.1016/S0140-6736(07)60983-0 17586306

[b7] Øra I. & Eggert A. Progress in treatment and risk stratification of neuroblastoma: impact on future clinical and basic research. Semin Cancer Biol 21, 217–28 (2011). 21798350 10.1016/j.semcancer.2011.07.002

[b8] Reynolds C. P., Matthay K. K., Villablanca J. G. & Maurer B. J. Retinoid therapy of high-risk neuroblastoma. Cancer Lett. 197, 185–92 (2003). 12880980 10.1016/s0304-3835(03)00108-3

[b9] Cheung N. K. . Murine anti-GD2 monoclonal antibody 3F8 combined with granulocyte-macrophage colony-stimulating factor and 13-cis-retinoic acid in high-risk patients with stage 4 neuroblastoma in first remission. J Clin Oncol. 30, 3264–70 (2012). 22869886 10.1200/JCO.2011.41.3807 PMC3434986

[b10] Reynolds C. P. . Retinoic-acid-resistant neuroblastoma cell lines show altered MYC regulation and high sensitivity to fenretinide. Med Pediatr Oncol. 35, 597–602 (2000). 11107126 10.1002/1096-911x(20001201)35:6<597::aid-mpo23>3.0.co;2-b

[b11] Armstrong J. L., Redfern C. P. & Veal G. J. 13-cis retinoic acid and isomerisation in paediatric oncology–is changing shape the key to success? Biochem Pharmacol. 69, 1299–306 (2005). 15826600 10.1016/j.bcp.2005.02.003

[b12] Ghattass K., Assah R., El-Sabban M. & Gali-Muhtasib H. Targeting hypoxia for sensitization of tumors to radio- and chemotherapy. Curr Cancer Drug Targets. 13, 670–85 (2013). 23687923 10.2174/15680096113139990004

[b13] Burroughs S. K. . Hypoxia inducible factor pathway inhibitors as anticancer therapeutics. Future Med Chem. 5, 553–72 (2013). 23573973 10.4155/fmc.13.17 PMC3871878

[b14] Sandau K. B., Fandrey J. & Brüne B. Accumulation of HIF-1alpha under the influence of nitric oxide. Blood 97, 1009–15 (2001). 11159530 10.1182/blood.v97.4.1009

[b15] Hellwig-Bürgel T., Rutkowski K., Metzen E., Fandrey J. & Jelkmann W. Interleukin-1beta and tumor necrosis factor-alpha stimulate DNA binding of hypoxia-inducible factor-1. Blood 94, 1561–7 (1999). 10477681

[b16] Feldser D. . Reciprocal positive regulation of hypoxia-inducible factor 1alpha and insulin-like growth factor 2. Cancer Res 59, 3915–8 (1999). 10463582

[b17] Jiang B. H., Agani F., Passaniti A. & Semenza G. L. V-SRC induces expression of hypoxia-inducible factor 1 (HIF-1) and transcription of genes encoding vascular endothelial growth factor and enolase 1: involvement of HIF-1 in tumor progression. Cancer Res 57, 5328–35 (1997). 9393757

[b18] Ravi R. . Regulation of tumor angiogenesis by p53-induced degradation of hypoxia-inducible factor 1alpha. Genes Dev 14, 34–44 (2000). 10640274 PMC316350

[b19] Zundel W. . Loss of PTEN facilitates HIF-1-mediated gene expression. Genes Dev 14, 391–6 (2000). 10691731 PMC316386

[b20] Hartwich J. . HIF-1α activation mediates resistance to anti-angiogenic therapy in neuroblastoma xenografts. J Pediatr Surg. 48, 39–46 (2013). 23331791 10.1016/j.jpedsurg.2012.10.016 PMC3601778

[b21] Puppo M. . Topotecan inhibits vascular endothelial growth factor production and angiogenic activity induced by hypoxia in human neuroblastoma by targeting hypoxia-inducible factor-1alpha and -2alpha. Mol Cancer Ther. 7, 1974–84 (2008). 18645007 10.1158/1535-7163.MCT-07-2059

[b22] Axelson H., Fredlund E., Ovenberger M., Landberg G. & Påhlman S. Hypoxia-induced dedifferentiation of tumor cells-a mechanism behind heterogeneity and aggressiveness of solid tumors. Semin Cell Dev Biol. 16, 554–63 (2005). 16144692 10.1016/j.semcdb.2005.03.007

[b23] Jögi A. . Human neuroblastoma cells exposed to hypoxia: induction of genes associated with growth, survival, and aggressive behavior. Exp Cell Res. 295, 469–87 (2004). 15093745 10.1016/j.yexcr.2004.01.013

[b24] Edsjö A., Holmquist L. & Påhlman S. Neuroblastoma as an experimental model for neuronal differentiation and hypoxia-induced tumor cell dedifferentiation. Semin Cancer Biol. 17, 248–56 (2007). 16828305 10.1016/j.semcancer.2006.04.005

[b25] Merrill R. A. . All-trans retinoic acid-responsive genes identified in the human SH-SY5Y neuroblastoma cell line and their regulated expression in the nervous system of early embryos. Biol Chem. 385, 605–14 (2004). 15318809 10.1515/BC.2004.075

[b26] Chambaut-Guérin A. M., Hérigault S., Rouet-Benzineb P., Rouher C. & Lafuma C. Induction of matrix metalloproteinase MMP-9 (92-kDa gelatinase) by retinoic acid in human neuroblastoma SKNBE cells: relevance to neuronal differentiation. J Neurochem. 74, 508–17 (2000). 10646501 10.1046/j.1471-4159.2000.740508.x

[b27] Reynolds C. P. Differentiating agents in pediatric malignancies: retinoids in neuroblastoma. Curr Oncol Rep. 2, 511–8 (2000). 11122886 10.1007/s11912-000-0104-y

[b28] Conrad P. W., Freeman T. L., Beitner-Johnson D. & Millhorn D. E. EPAS1 trans-activation during hypoxia requires p42/p44 MAPK. J Biol Chem 274, 33709–13 (1999). 10559262 10.1074/jbc.274.47.33709

[b29] Luciani P. . Exendin-4 induces cell adhesion and differentiation and counteracts the invasive potential of human neuroblastoma cells. PLoS One 8, (2013). 10.1371/journal.pone.0071716 PMC3750033 23990978

[b30] Celay J. . Changes in gene expression profiling of apoptotic genes in neuroblastoma cell lines upon retinoic acid treatment. PLoS One. 8, e62771 (2013). 23650528 10.1371/journal.pone.0062771 PMC3641123

[b31] Marstrand T. T. . A conceptual framework for the identification of candidate drugs and drug targets in acute promyelocytic leukemia. Leukemia 24, 1265–75 (2010). 20508621 10.1038/leu.2010.95

[b32] Coltella N. . HIF factors cooperate with PML-RARα to promote acute promyelocytic leukemia progression and relapse. EMBO Mol Med. 6, 640–50 (2014). 24711541 10.1002/emmm.201303065 PMC4023886

[b33] Meller K. Gradient isolation of glial cells: evidence that flat epithelial cells are astroglial cell precursors. Cell Tissue Res. 249, 79–88 (1987). 3621297 10.1007/BF00215421

[b34] Dimri G. P. . A biomarker that identifies senescent human cells in culture and in aging skin *in vivo*. Proc Natl Acad Sci USA 92, 9363–7 (1995). 7568133 10.1073/pnas.92.20.9363 PMC40985

[b35] Mossé Y. P. . Neuroblastoma in older children, adolescents and young adults: a report from the International Neuroblastoma Risk Group project. Pediatr Blood Cancer. 61, 627–35 (2014). 24038992 10.1002/pbc.24777

[b36] Matthay K. K. . Treatment of high-risk neuroblastoma with intensive chemotherapy, radiotherapy, autologous bone marrow transplantation, and 13-cis-retinoic acid. Children’s Cancer Group. N Engl J Med. 341, 1165–73 (1999). 10519894 10.1056/NEJM199910143411601

[b37] Giaccone G. & Pinedo H. M. Drug Resistance. Oncologist. 1, 82–87 (1996). 10387972

[b38] Hussein D., Estlin E. J., Dive C. & Makin G. W. Chronic hypoxia promotes hypoxia-inducible factor-1alpha-dependent resistance to etoposide and vincristine in neuroblastoma cells. Mol Cancer Ther. 5, 2241–50 (2006). 16985058 10.1158/1535-7163.MCT-06-0145

[b39] Wang D. . Hypoxia promotes etoposide (VP-16) resistance in neuroblastoma CHP126 cells. Pharmazie. 65, 51–6 (2010). 20187579

[b40] Pietras A. . HIF-2alpha maintains an undifferentiated state in neural crest-like human neuroblastoma tumor-initiating cells. Proc Natl Acad Sci USA. 106, 16805–10 (2009). 19805377 10.1073/pnas.0904606106 PMC2745331

[b41] Raval R. R. . Contrasting properties of hypoxia-inducible factor 1 (HIF-1) and HIF-2 in von Hippel-Lindau-associated renal cell carcinoma. Mol Cell Biol. 25, 5675–86 (2005). 15964822 10.1128/MCB.25.13.5675-5686.2005 PMC1157001

[b42] Gunnur Dikmen Z. . *In vivo* and *in vitro* Effects of a HIF-1a Inhibitor, RX-004. Journal of Cellular Biochemistry 104, 985–994 (2008). 18275063 10.1002/jcb.21681 PMC3375689

[b43] Fraisl P., Aragonés J., Carmeliet P. Inhibition of oxygen sensors as a therapeutic strategy for ischaemic and inflammatory disease. Nature Reviews Drug Discovery 8, 139–152 (2009). 19165233 10.1038/nrd2761

[b44] Jeong W. . Pilot trial of EZN-2968, an antisense oligonucleotide inhibitor of hypoxia-inducible factor-1 alpha (HIF-1α), in patients with refractory solid tumors. Cancer Chemother Pharmacol. 73, 343–8 (2014). 24292632 10.1007/s00280-013-2362-z PMC8375568

[b45] Kummar S. . Multihistology, target-driven pilot trial of oral topotecan as an inhibitor of hypoxia-inducible factor-1α in advanced solid tumors. Clin Cancer Res. 17, 5123–31 (2011). 21673063 10.1158/1078-0432.CCR-11-0682 PMC3149769

[b46] Onnis B., Rapisarda A. & Melillo G. Development of HIF-1 inhibitors for cancer therapy. J Cell Mol Med. 13, 2780–6 (2009). 19674190 10.1111/j.1582-4934.2009.00876.x PMC2832082

[b47] Watson J. A., Watson C. J., McCann A. & Baugh J. Epigenetics, the epicenter of the hypoxic response. Epigenetics. 5, 293–6 (2010). 20418669 10.4161/epi.5.4.11684

[b48] Poljaková J. . Hypoxia-mediated histone acetylation and expression of N-myc transcription factor dictate aggressiveness of neuroblastoma cells. Oncol Rep. 31, 1928–34 (2014). 24481548 10.3892/or.2014.2999

[b49] Acosta S. . Comprehensive characterization of neuroblastoma cell line subtypes reveals bilineage potential similar to neural crest stem cells. BMC Dev Biol. 9–12 (2009). 19216736 10.1186/1471-213X-9-12 PMC2647534

[b50] Zhu H. . Effect of hypoxia/reoxygenation on cell viability and expression and secretion of neurotrophic factors (NTFs) in primary cultured schwann cells. Anat Rec (Hoboken). 293, 865–70 (2010). 20186961 10.1002/ar.21105

[b51] Chlenski A. . Anti-angiogenic SPARC peptides inhibit progression of neuroblastoma tumors. Mol Cancer. 9, 138 (2010). 20525313 10.1186/1476-4598-9-138 PMC2895596

[b52] Park S. H., Lim J. S. & Jang K. L. All-trans retinoic acid induces cellular senescence via upregulation of p16, p21, and p27. Cancer Lett. 310, 232–9 (2011). 21803488 10.1016/j.canlet.2011.07.009

[b53] Kilic Eren M. & Tabor V. The role of hypoxia inducible factor-1 alpha in bypassing oncogene-induced senescence. PLoS One. 9, e101064 (2014). 24984035 10.1371/journal.pone.0101064 PMC4077769

[b54] Clark O., Daga S. & Stoker A. W. Tyrosine phosphatase inhibitors combined with retinoic acid can enhance differentiation of neuroblastoma cells and trigger ERK- and AKT-dependent, p53-independent senescence. Cancer Lett. 328, 44–54 (2013). 23022267 10.1016/j.canlet.2012.09.014

[b55] Liu S. . ‘Cross-talk’ between Schwannian stroma and neuroblasts promotes neuroblastoma tumor differentiation and inhibits angiogenesis. Cancer Lett. 228, 125–31 (2005). 15935552 10.1016/j.canlet.2005.01.056

[b56] Molenaar J. . Copy number defects of G1-cell cycle genes in neuroblastoma are frequent and correlate with high expression of E2F target genes and a poor prognosis. Genes Chromosomes Cancer. 1, 10–9 (2012). 10.1002/gcc.20926 22034077

[b57] Benjamini Y. Discovering the false discovery rate. J. R. Statist. Soc. 72, 405–416 (2010).

[b58] Zhang B. . The significance of controlled conditions in lentiviral vector titration and in the use of multiplicity of infection (MOI) for predicting gene transfer events. Genet Vaccines Ther. 2, 6 (2004). 15291957 10.1186/1479-0556-2-6 PMC514534

[b59] Livak K. J. & Schmittgen T. D. Analysis of relative gene expression data using real-time quantitative PCR and the 2^-ΔΔCT^ Method. Methods. 25, 402–8 (2001). 11846609 10.1006/meth.2001.1262

[b60] Dickinson M. E. . Sensitive imaging of spectrally overlapping fluorochromes using the LSM 510 META. Multiphoton Microscopy in the Biomedical Sciences II, 10.1117/12.470686 (2002).

