# Supplementary material for: Inhibition of hypoxia inducible factors combined with all-trans retinoic acid treatment enhances glial transdifferentiation of neuroblastoma cells
Source: Sci Rep. 2015 Jun 9;5:11158. doi: 10.1038/srep11158 (PMC4460899; doi:10.1038/srep11158)
Supplement: Supplementary Information [file srep11158-s1.doc]

**Inhibition of hypoxia inducible factors combined with all-*trans* retinoic acid treatment enhances glial transdifferentiation of neuroblastoma cells**

Flora Cimmino^1, 2^, Lucia Pezone^2, 3^, Marianna Avitabile^1, 2^, Giovanni Acierno^1, 2^, Immacolata Andolfo^1, 2^, Mario Capasso^1, 2^ and Achille Iolascon^1,2^,*

^1^ Dipartimento di Medicina Molecolare e Biotecnologie Mediche, Università degli studi di Napoli “Federico II”, Naples, Italy

^2^ CEINGE Biotecnologie Avanzate, Naples, Italy

^3^ Scuola di Medicina e Chirurgia, Università degli studi di Verona, Verona, Italy

***Corresponding Author: Achille Iolascon.**

Dipartimento di Medicina Molecolare e Biotecnologie Mediche

Università degli Studi di Napoli “Federico II”

CEINGE Biotecnologie Avanzate

Via G. Salvatore 482

Naples 80145, Italy

Tel: +39-081-3737897

Fax: +39-081-3737804

Email: achille.[iolascon@unina.it](mailto:iolascon@unina.it)

**Supplementary Data**

**
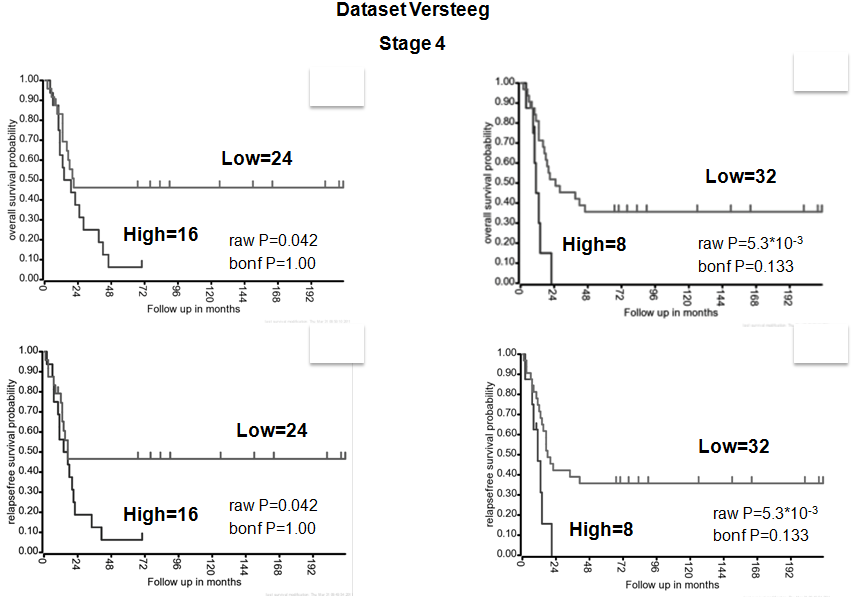
**

**Figure S1. *HIF1A* and *EPAS1* expression is associated with poor survival for patients with stage 4 NBL.** Kaplan-Maier analysis with patients grouped according to the optimal cut-off (calculated using the R2 web tool) in the expression of *HIF1A* and *EPAS1* for overall survival and relapse-free survival for 40 patients with stage 4 NBL (Versteeg dataset). The “raw P” indicates the uncorrected p-value, and the “bonf P” indicates the p-value corrected for multiple tests according to the Bonferroni method.


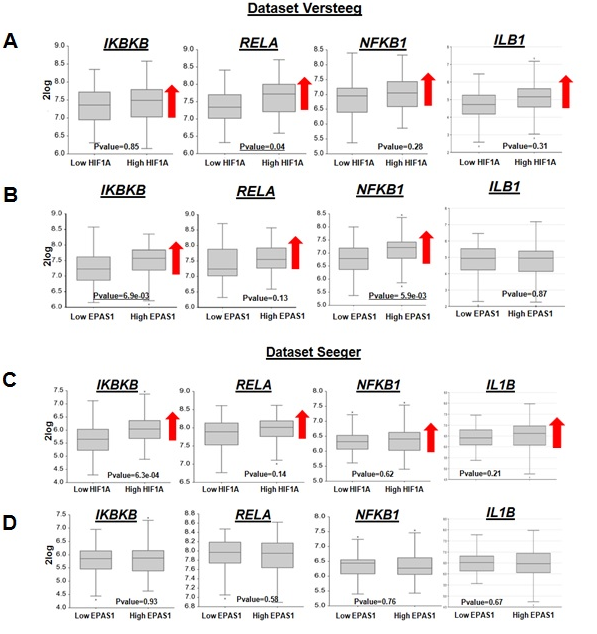


**Figure S2. *IKBKB*, *RELA*, *NFKB1* and *ILB1* expression in the subgroups of patient with NBL**. Box plots showing the expression levels (Log2) of *IKBKB*, *RELA*, *NFKB1* and *ILB1* in the patient subgroups of ‘Low’ *HIF1A* and ‘High’ *HIF1A* expression in the Versteeg (A) and Seeger (C) datasets, and in the ‘Low’ *EPAS1* and ‘High’ EPAS1 expression in the Versteeg (B) and Seeger (D) datasets.


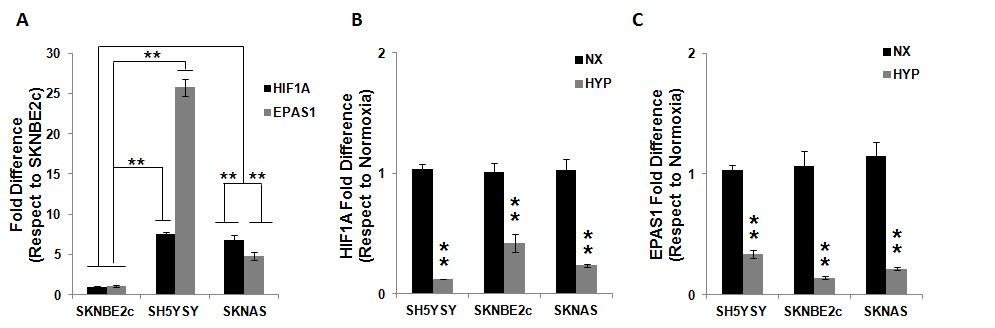


**Figure S3. mRNA expression of *HIF1A* and *EPAS1* in NBL cells.** (A) Expression of *HIF1A* and *EPAS1* as evaluated by RT-PCR in the SHSY5Y, SKNBE2c, and SKNAS cell lines. The data are shown as fold-differences among the three cell lines. The mean fold change of 2^-(average ∆∆CT)^ was determined using the mean differences in the ∆C_T_ between the *HIF1A* and *EPAS1* expression in the SHSY5Y and SKNAS cells and the ∆C_T_ *HIF1A* and *EPAS1* expression in the SKNBE2c cells (as internal control). (B, C) The SHSY5Y, SKNBE2c, and SKNAS cell lines were grown under normoxia (NX) and hypoxia (HYP) (1% oxygen for 6 h). The fold differences of *HIF1A* (B) and *EPAS1* (C) expression under hypoxia with respect to normoxia were calculated as folds changes of 2^-(average ∆∆CT)^. The fold changes were determined using the mean differences in the ∆C_T_ between the gene expression under hypoxia and the ∆C_T_ for gene expression under normoxia (as internal control) for each cell line. Data are means of three experiments (* P ≤0.05; ** P ≤0.01).


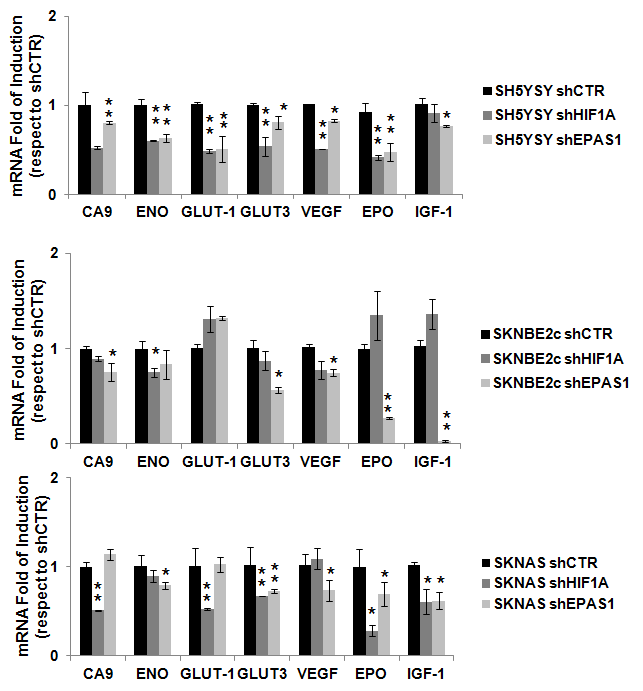


**Figure S4. mRNA expression under *HIF1A* and *EPAS1* induction.** The *HIF1A* and *EPAS1* induction of the expression of *CA9*, *ENO*, *GLUT-1*, *GLUT-3*, *VEGF*, *EPO*, *IGF-2* was evaluated by RT-PCR in the SHSY5Y (A), SKNBE2c (B) and SKNAS (C) cell lines previously silenced for *HIF1A* or *EPAS1* expression (i.e., shHIF1A or shEPAS1, respectively). The data are fold-changes of induction with respect to the shCTR unsilenced cells. The mean fold change of 2^-(average ∆∆CT)^ was determined using the mean difference in the ∆C_T_ between the gene expression in the shHIF1A or shEPAS1cells and the ∆C_T_ for gene expression in the shCTR cells (as internal control). Data are means of three experiments (* P ≤0.05; ** P ≤0.01). The gene-specific primers were designed using the PRIMEREXPRESS software (Applied Biosystems), as: CA9 Forward (F) TGCTAAGCAGCTCCACACCC, Reverse (R) TGCGTCGCTCGGAAGTTC; ENO (F) (R) ; GLUT-1 (F) CCTGCTCATCAACCGCAAC, (R) TCATGGGTCACGTCAGCTGT; GLUT-3 (F) CCAGCTGGGCATCGTTG, (R) CGGCCATAGCTCTTCAGACC; VEGF (F) (R); EPO (F) (R); IGF-1 (F) TCCTGGAGACGTACTGTGCT (R) CTGGGGSSGTTGTCCGGAAG.


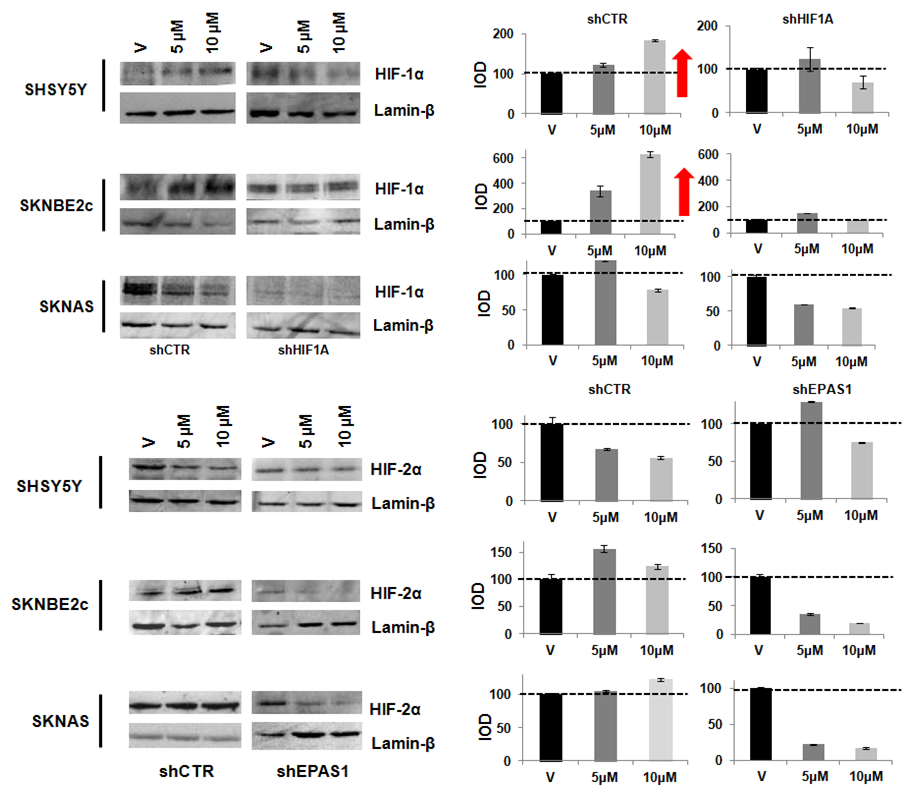


**Figure S5. HIF-1α and HIF-2α** **protein expression upon ATRA treatment.** SHSY5Y, SKNBE2c and SKNAS shCTR, shHIF1A, and shEPAS1 cells were treated with 5 µM or 10 µM ATRA or vehicle (V) for 6 days. (A) Western blotting shows HIF-1α protein levels (A) and HIF-2α protein levels upon ATRA treatment. Bar graphs show the integral optical density (IOD) for each band normalized respect to lamin-β expression. The IOD are expressed in percentages with respect to vehicle treated cells.


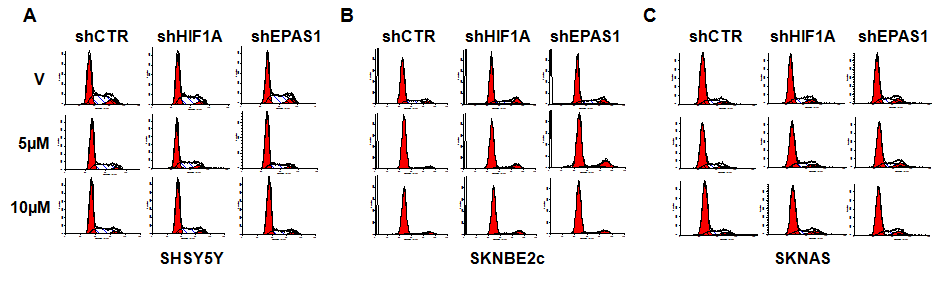


**Figure S6. ATRA treatment: cell cycle.** Unsilenced cells (shCTR) and *HIF1A* or *EPAS1* silenced cells (i.e., shHIF1A or shEPAS1, respectively) treated for 6 days with 5 µM and 10 µM ATRA and then processed for flow cytometry using propidium iodide. Flow-histograms are shown for the SHSY5Y (A), SKNBE2c (B), SKNAS (C) cells.

**Supplementary Table S1.** Differential gene expression between ‘High’ and ‘Low’ *EPAS1* mRNA expression levels.

| **Versteeg dataset** | | |  | **Seeger dataset** | | |
| --- | --- | --- | --- | --- | --- | --- |
| **Gene** | **R-value** | **p-value** |  | **Gene** | **R-value** | **p-value** |
| *UNC5C* | 0.494 | 0.0007 |  | *CITED2* | 0.598 | 1.65E-08 |
| *KDR* | 0.396 | 0.0381 |  | *SH3GL3* | -0.473 | 0.0002 |
| *EYA4* | 0.396 | 0.0464 |  | *EYA4* | 0.462 | 0.0003 |
| *GPC3* | 0.383 | 0.0532 |  | *SFRP1* | 0.433 | 0.0012 |
| *GNA13* | -0.400 | 0.0535 |  | *INSM1* | 0.418 | 0.0018 |
| *LAMA5* | 0.373 | 0.0604 |  | *NRG1* | -0.420 | 0.0019 |
| *ESRRG* | -0.369 | 0.0635 |  | *SMAD1* | 0.407 | 0.0025 |
| *EDA2R* | -0.373 | 0.0688 |  | *DLK1* | 0.408 | 0.0028 |
| *TRPC5* | -0.354 | 0.0711 |  | *LY6H* | -0.380 | 0.0085 |
| *TNFAIP1* | 0.356 | 0.0720 |  | *GREM1* | 0.372 | 0.0112 |
| *SIX5* | 0.335 | 0.0721 |  | *DCX* | -0.363 | 0.0154 |
| *LAMA3* | 0.340 | 0.0727 |  | *GPR56* | -0.357 | 0.0164 |
| *LAMB1* | 0.339 | 0.0730 |  | *CYP46A1* | -0.358 | 0.0164 |
| *GATA2* | 0.358 | 0.0733 |  | *SEMA3D* | -0.359 | 0.0167 |
| *HAND1* | 0.360 | 0.0737 |  | *NELL1* | 0.343 | 0.0273 |
| *ANGPTL2* | 0.335 | 0.0740 |  | *SEMA3A* | -0.337 | 0.0312 |
| *LRP5* | 0.337 | 0.0749 |  | *POU4F1* | 0.338 | 0.0318 |
| *ST8SIA4* | -0.340 | 0.0756 |  | *PAK3* | -0.333 | 0.0328 |
| *ELN* | 0.362 | 0.0758 |  | *SMPD1* | -0.334 | 0.0333 |
| *TIMM8A* | -0.335 | 0.0758 |  | *NGFRAP1* | -0.329 | 0.0347 |
| *TLL2* | -0.332 | 0.0759 |  | *POU4F2* | 0.330 | 0.0357 |
| *WIF1* | -0.341 | 0.0761 |  | *ITGA8* | -0.327 | 0.0366 |
| *SPP1* | -0.342 | 0.0795 |  | *HECA* | 0.320 | 0.0461 |
| *ETS2* | 0.343 | 0.0796 |  | *SMPD3* | -0.318 | 0.0470 |
| *DLL1* | 0.344 | 0.0799 |  | *IL18* | -0.313 | 0.0510 |
| *TFAP2B* | 0.349 | 0.0821 |  | *UNC5C* | 0.314 | 0.0517 |
| *EBF4* | 0.344 | 0.0843 |  | *DKK1* | 0.314 | 0.0531 |
| *FOXO1* | 0.346 | 0.0858 |  | *PKD2* | 0.308 | 0.0587 |
| *EMD* | -0.323 | 0.0904 |  | *CHODL* | 0.307 | 0.0593 |
| *ACVRL1* | 0.324 | 0.0920 |  | *CDH11* | -0.306 | 0.0604 |
| *ENG* | 0.325 | 0.0924 |  | *TBR1* | -0.304 | 0.0615 |
| *SEMA3D* | -0.325 | 0.0955 |  | *SLIT3* | 0.302 | 0.0636 |
| *INSM1* | 0.319 | 0.0989 |  | *DDX47* | 0.302 | 0.0653 |
| *ACVR1* | 0.315 | 0.1001 |  | *SPOCK2* | -0.295 | 0.0768 |
| *NPR3* | 0.319 | 0.1006 |  | *SEMA6D* | -0.294 | 0.0773 |
| *MYH9* | 0.317 | 0.1015 |  | *PAPSS1* | 0.292 | 0.0778 |
| *OPHN1* | -0.316 | 0.1017 |  | *EXT2* | -0.295 | 0.0787 |
| *SFRP1* | 0.316 | 0.1033 |  | *TRPC5* | -0.292 | 0.0798 |
| *TSHZ1* | 0.312 | 0.1099 |  | *HDAC9* | 0.290 | 0.0807 |
| *PRKAR1A* | -0.306 | 0.1142 |  | *LAMB1* | 0.288 | 0.0838 |
| *AGT* | 0.308 | 0.1150 |  | *ZEB2* | 0.286 | 0.0899 |
| *COL18A1* | 0.305 | 0.1151 |  | *PLXNA3* | -0.282 | 0.0986 |
| *SIX3* | -0.306 | 0.1161 |  | *KALRN* | -0.277 | 0.1143 |
| *FZD4* | 0.308 | 0.1172 |  | *HRAS* | -0.276 | 0.1158 |
| *BEX1* | -0.309 | 0.1174 |  | *FGF14* | 0.273 | 0.1233 |
| *EPHB4* | 0.307 | 0.1178 |  | *ETS2* | 0.273 | 0.1256 |
| *PKD2* | 0.302 | 0.1233 |  | *PHGDH* | 0.268 | 0.1265 |
| *KCNQ2* | 0.301 | 0.1262 |  | *BTG1* | 0.268 | 0.1269 |
| *FGFR3* | 0.300 | 0.1284 |  | *DHCR24* | -0.269 | 0.1278 |
| *LFNG* | 0.298 | 0.1322 |  | *PSME4* | 0.267 | 0.1285 |
| *SPEG* | 0.296 | 0.1380 |  | *ROBO3* | 0.271 | 0.1287 |
| *AGPAT6* | 0.291 | 0.1421 |  | *HHAT* | 0.270 | 0.1295 |
| *FLII* | 0.291 | 0.1438 |  | *CMKLR1* | -0.269 | 0.1296 |
| *NGRN* | -0.294 | 0.1440 |  | *STMN1* | -0.258 | 0.1306 |
| *HRAS* | -0.290 | 0.1443 |  | *RELN* | 0.265 | 0.1313 |
| *SOSTDC1* | -0.291 | 0.1460 |  | *AES* | 0.269 | 0.1315 |
| *COL4A2* | 0.286 | 0.1461 |  | *CCL2* | -0.259 | 0.1321 |
| *NOTCH3* | 0.293 | 0.1462 |  | *GAL* | 0.265 | 0.1323 |
| *SLIT3* | 0.292 | 0.1473 |  | *TWIST1* | 0.259 | 0.1328 |
| *STAB1* | 0.286 | 0.1483 |  | *SEMA6A* | -0.259 | 0.1335 |
| *LSR* | 0.286 | 0.1492 |  | *SERPINI1* | 0.264 | 0.1335 |
| *ERG* | 0.292 | 0.1494 |  | *SMARCA1* | -0.263 | 0.1336 |
| *ADAM18* | -0.284 | 0.1494 |  | *ACVR1* | 0.259 | 0.1343 |
| *GJA5* | 0.287 | 0.1497 |  | *NME5* | -0.261 | 0.1350 |
| *DLL4* | 0.284 | 0.1503 |  | *KLF3* | 0.260 | 0.1353 |
| *SMAD1* | 0.288 | 0.1504 |  | *PBXIP1* | 0.261 | 0.1363 |
| *COL15A1* | 0.287 | 0.1507 |  | *COL9A2* | -0.256 | 0.1370 |
| *TSHZ3* | 0.287 | 0.1520 |  | *NGFR* | -0.260 | 0.1372 |
| *ERC2* | -0.281 | 0.1521 |  | *MARK2* | -0.262 | 0.1372 |
| *DAB2* | 0.283 | 0.1529 |  | *NLGN1* | 0.261 | 0.1375 |
| *ENPEP* | 0.281 | 0.1534 |  | *PHYH* | 0.256 | 0.1378 |
| *FRZB* | 0.280 | 0.1541 |  | *EYA1* | 0.254 | 0.1425 |
| *FZD8* | 0.282 | 0.1546 |  | *PRKRA* | 0.253 | 0.1446 |
| *SEMA5A* | 0.281 | 0.1554 |  | *TCF12* | 0.253 | 0.1448 |
| *ANGPT1* | 0.278 | 0.1613 |  | *GRSF1* | 0,252 | 0.1465 |
| *PRELP* | 0.278 | 0.1618 |  | *TRIM3* | -0.251 | 0.1495 |
| *NPR1* | 0.277 | 0.1631 |  | *TLE2* | 0.249 | 0.1504 |
| *BDNF* | -0.276 | 0.1634 |  | *SEMA4D* | 0.250 | 0.1512 |
| *TBX2* | -0.272 | 0.1840 |  | *CTNND2* | -0.249 | 0.1523 |
| *SNCA* | -0.271 | 0.1877 |  | *CDK5RAP3* | -0.248 | 0.1544 |
| *ZIC2* | -0.270 | 0.1879 |  | *SLIT2* | -0.246 | 0.1609 |
| *SVIL* | 0.270 | 0.1896 |  | *SKIL* | -0.246 | 0.1626 |
| *NRP1* | 0.268 | 0.1897 |  | *SEPP1* | -0.244 | 0.1679 |
| *ST8SIA2* | 0.268 | 0.1916 |  | *RTN1* | -0.243 | 0.1710 |
| *SOX9* | -0.269 | 0.1919 |  | *CDKN1B* | 0.243 | 0.1723 |
| *HOXC9* | 0.267 | 0.1944 |  | *GAP43* | -0.241 | 0.1766 |
| *PLXND1* | 0.264 | 0.2080 |  | *GADD45G* | -0.241 | 0.1770 |
| *FLNA* | 0.264 | 0.2101 |  | *PFN1* | 0.239 | 0.1832 |
| *MPPED2* | -0.254 | 0.2170 |  | *VCAN* | -0.238 | 0.1837 |
| *WFS1* | 0.255 | 0.2183 |  | *IL7* | -0.238 | 0.1840 |
| *SEMA4C* | 0.254 | 0.2185 |  | *PPARD* | 0.236 | 0.1910 |
| *ID1* | 0.260 | 0.2186 |  | *JMJD6* | -0.235 | 0.1946 |
| *HSPB2* | 0.261 | 0.2186 |  | *UGDH* | 0.235 | 0.1951 |
| *ZEB2* | 0.260 | 0.2187 |  | *CHRM3* | 0.234 | 0.1965 |
| *MMP2* | 0.261 | 0.2192 |  | *STMN2* | -0.234 | 0.1979 |
| *VEZF1* | -0.260 | 0.2193 |  | *PTPRR* | 0.233 | 0.1991 |
| *POFUT1* | 0.255 | 0.2199 |  | *MYH6* | -0.232 | 0.1998 |
| *TUBD1* | -0.257 | 0.2215 |  | *SPRY2* | -0.232 | 0.2002 |
| *SNTG2* | 0.255 | 0.2218 |  | *TFIP11* | 0.231 | 0.2017 |
| *ELK3* | 0.250 | 0.2220 |  | *TP53BP2* | 0.231 | 0.2048 |
| *FEZ1* | -0.256 | 0.2223 |  | *PAPSS2* | 0.226 | 0.2286 |
| *COL6A3* | 0.255 | 0.2227 |  | *FGF9* | -0.226 | 0.2293 |
| *HDAC4* | 0.255 | 0.2228 |  | *ACP2* | -0.225 | 0.2329 |
| *GRSF1* | 0.256 | 0.2232 |  | *HBEGF* | -0.224 | 0.2332 |
| *JAG1* | 0.258 | 0.2234 |  | *PCOLCE* | 0.223 | 0.2386 |
| *MAML3* | 0.250 | 0.2236 |  | *PPP1R9A* | -0.223 | 0.2404 |
| *MYH11* | 0.257 | 0.2237 |  | *TLX3* | 0.221 | 0.2491 |
| *APAF1* | 0.258 | 0.2239 |  | *BRAF* | -0.221 | 0.2503 |
| *FGF5* | -0.256 | 0.2240 |  | *NUMB* | -0.219 | 0.2552 |
| *CITED2* | 0.256 | 0.2243 |  | *CREG1* | -0.218 | 0.2559 |
| *SKI* | 0.250 | 0.2246 |  | *SEMA3E* | -0.218 | 0.2572 |
| *SIM2* | -0.251 | 0.2250 |  | *SH2B3* | -0.217 | 0.2573 |
| *THY1* | 0.257 | 0.2256 |  | *IFRD1* | -0.217 | 0.2590 |
| *PPARD* | 0.250 | 0.2257 |  | *DIXDC1* | 0.218 | 0.2594 |
| *SOX11* | -0.250 | 0.2259 |  | *LEFTY2* | -0.217 | 0.2602 |
| *PBXIP1* | 0.251 | 0.2268 |  | *CACNB2* | -0.215 | 0.2606 |
| *AR* | 0.251 | 0.2271 |  | *NEO1* | -0.215 | 0.2623 |
| *MLF1* | -0.252 | 0.2278 |  | *KRT19* | -0.215 | 0.2628 |
| *ITGB5* | 0.250 | 0.2278 |  | *PNMA1* | -0.216 | 0.2635 |
| *FOXK1* | 0.251 | 0.2286 |  | *TIMP1* | -0.213 | 0.2677 |
| *LMO1* | 0.246 | 0.2315 |  | *NES* | 0.213 | 0.2682 |
| *PLXNA1* | 0.245 | 0.2328 |  | *DDX41* | 0.214 | 0.2690 |
| *DLK1* | 0.247 | 0.2328 |  | *PAX9* | 0.212 | 0.2723 |
| *NPTN* | -0.246 | 0.2333 |  | *CALCA* | -0.210 | 0.2854 |
| *TBX5* | -0.245 | 0.2335 |  | *MFN2* | -0.210 | 0.2865 |
| *UNC5B* | 0.246 | 0.2341 |  | *DACH1* | 0.208 | 0.2924 |
| *SRF* | 0.245 | 0.2341 |  | *TRO* | -0.208 | 0.2954 |
| *TSHZ2* | 0.247 | 0.2343 |  | *THRA* | -0.207 | 0.2971 |
| *HTN3* | -0.245 | 0.2345 |  | *TPP1* | -0.207 | 0.2976 |
| *HEYL* | 0.247 | 0.2350 |  | *NRP2* | -0.207 | 0.2994 |
| *SPATA18* | -0.243 | 0.2436 |  |  |  |  |
| *NES* | 0.242 | 0.2439 |  |  |  |  |
| *NELL1* | 0.241 | 0.2450 |  |  |  |  |
| *ADAM10* | 0.241 | 0.2452 |  |  |  |  |
| *HOXD8* | -0.242 | 0.2455 |  |  |  |  |
| *FLNB* | 0.242 | 0.2471 |  |  |  |  |
| *DGCR2* | 0.240 | 0.2492 |  |  |  |  |
| *SHC3* | -0.240 | 0.2500 |  |  |  |  |
| *EYA1* | 0.239 | 0.2544 |  |  |  |  |
| *LHX6* | 0.238 | 0.2561 |  |  |  |  |
| *MEA1* | -0.237 | 0.2562 |  |  |  |  |
| *PAPSS2* | 0.237 | 0.2573 |  |  |  |  |
| *SPARC* | 0.237 | 0.2580 |  |  |  |  |
| *NGFRAP1* | -0.236 | 0.2611 |  |  |  |  |
| *POU4F2* | 0.235 | 0.2611 |  |  |  |  |
| *AHNAK* | 0.234 | 0.2612 |  |  |  |  |
| *ROBO4* | 0.235 | 0.2623 |  |  |  |  |
| *KLK6* | -0.234 | 0.2627 |  |  |  |  |
| *QKI* | -0.236 | 0.2627 |  |  |  |  |
| *DVL3* | 0.233 | 0.2635 |  |  |  |  |
| *COL1A1* | 0.236 | 0.2638 |  |  |  |  |
| *ITGA11* | 0.232 | 0.2641 |  |  |  |  |
| *RREB1* | 0.234 | 0.2645 |  |  |  |  |
| *FGF10* | -0.233 | 0.2652 |  |  |  |  |
| *NTNG1* | -0.232 | 0.2653 |  |  |  |  |
| *IGFBP4* | 0.232 | 0.2656 |  |  |  |  |
| *CCK* | -0.232 | 0.2665 |  |  |  |  |
| *TRAF4* | -0.232 | 0.2669 |  |  |  |  |
| *ANPEP* | 0.228 | 0.2673 |  |  |  |  |
| *NR5A2* | 0.228 | 0.2674 |  |  |  |  |
| *TLE3* | 0.232 | 0.2684 |  |  |  |  |
| *SERPINE2* | 0.228 | 0.2687 |  |  |  |  |
| *AFF3* | 0.228 | 0.2688 |  |  |  |  |
| *COL3A1* | 0.229 | 0.2688 |  |  |  |  |
| *FABP7* | -0.229 | 0.2701 |  |  |  |  |
| *SNAI2* | 0.229 | 0.2712 |  |  |  |  |
| *MYT1* | 0.230 | 0.2722 |  |  |  |  |
| *CHUK* | -0.229 | 0.2726 |  |  |  |  |
| *AEBP1* | 0.230 | 0.2736 |  |  |  |  |
| *SH3GL3* | -0.229 | 0.2736 |  |  |  |  |
| *CALCRL* | 0.229 | 0.2739 |  |  |  |  |
| *COL1A2* | 0.229 | 0.2752 |  |  |  |  |
| *POU3F3* | -0.226 | 0.2776 |  |  |  |  |
| *SLC30A1* | 0.226 | 0.2778 |  |  |  |  |
| *TGIF1* | 0.225 | 0.2811 |  |  |  |  |
| *VEGFC* | 0.225 | 0.2815 |  |  |  |  |
| *DLX2* | -0.224 | 0.2864 |  |  |  |  |
| *INSR* | 0.223 | 0.2887 |  |  |  |  |
| *ITGA2* | 0.223 | 0.2895 |  |  |  |  |
| *TCL1A* | 0.222 | 0.2916 |  |  |  |  |
| *TFIP11* | 0.222 | 0.2922 |  |  |  |  |
| *DKK2* | -0.221 | 0.2924 |  |  |  |  |
| *DCTN1* | 0.222 | 0.2925 |  |  |  |  |
| *SEMA5B* | 0.221 | 0.2931 |  |  |  |  |
| *DGKD* | 0.221 | 0.2955 |  |  |  |  |
| *DOPEY2* | 0.220 | 0.2978 |  |  |  |  |
| *MOV10* | 0.220 | 0.2989 |  |  |  |  |
| *DACH1* | 0.219 | 0.2995 |  |  |  |  |

**Supplementary Table 2.** Differential gene expression between ‘High’ and ‘Low’ *HIF1A* mRNA expression levels.

| **Versteeg dataset** | | |  | **Seeger dataset** | | |
| --- | --- | --- | --- | --- | --- | --- |
| **Gene** | **R-Value** | **P-value** |  | **Gene** | **R-value** | **p-value** |
| *JMJD6* | 0.582 | 0.00000 |  | *GMFB* | 0.396 | 0.0050 |
| *NTRK1* | -0.515 | 0.00020 |  | *KCNQ2* | -0.393 | 0.0051 |
| *ROR2* | 0.499 | 0.00035 |  | *RELN* | 0.397 | 0,0054 |
| *NRCAM* | -0.490 | 0.00036 |  | *PTHLH* | 0.400 | 0.0055 |
| *SPRED2* | 0.486 | 0.00038 |  | *ATXN3* | 0.389 | 0.0055 |
| *B3GNT5* | 0.492 | 0.00040 |  | *CNTFR* | -0.386 | 0.0059 |
| *HMX1* | -0.479 | 0.00046 |  | *APBA1* | -0.401 | 0.0060 |
| *SIX3* | 0.458 | 0.00125 |  | *HDAC5* | -0.383 | 0.0063 |
| *RNF113A* | 0.455 | 0.00135 |  | *NUMB* | 0.418 | 0.0063 |
| *INSRR* | -0.448 | 0.00168 |  | *FRAT2* | -0.379 | 0.0068 |
| *DLX6* | 0.439 | 0.00191 |  | *TCF25* | -0.376 | 0.0068 |
| *MAFF* | 0.440 | 0.00193 |  | *ZNF267* | 0.377 | 0.0071 |
| *PLXNC1* | -0.443 | 0.00195 |  | *BRSK2* | -0.402 | 0.0073 |
| *IL7* | -0.436 | 0.00203 |  | *MTR* | 0.370 | 0.0076 |
| *RAPGEF5* | -0.441 | 0.00205 |  | *C1GALT1* | 0.371 | 0.0076 |
| *SHC3* | 0.434 | 0.00212 |  | *CUL7* | -0.372 | 0.0076 |
| *VPRBP* | 0.425 | 0.00275 |  | *ATR* | 0.428 | 0.0077 |
| *APBA1* | -0.420 | 0.00281 |  | *DRD2* | -0.366 | 0.0081 |
| *NR2F1* | 0.425 | 0.00286 |  | *GLRB* | -0.405 | 0.0083 |
| *EIF2B2* | 0.426 | 0.00288 |  | *LIF* | 0.366 | 0.0084 |
| *ACHE* | -0.421 | 0.00290 |  | *APBA2* | -0.362 | 0.0089 |
| *TRERF1* | -0.421 | 0.00301 |  | *UGDH* | 0.362 | 0.0091 |
| *RET* | 0.421 | 0.00307 |  | *STMN3* | -0.360 | 0.0092 |
| *SOX6* | -0.412 | 0.00381 |  | *EGR3* | 0.357 | 0.0099 |
| *RBBP7* | 0.409 | 0.00382 |  | *CHUK* | 0.351 | 0.0116 |
| *GPI* | 0.409 | 0.00390 |  | *HMX1* | -0.351 | 0.0121 |
| *SCN8A* | -0.412 | 0.00394 |  | *SIAH1* | -0.352 | 0.0121 |
| *PRPS1* | 0.409 | 0.00405 |  | *SOX15* | -0.349 | 0.0122 |
| *SH2D2A* | -0.406 | 0.00422 |  | *ELF3* | 0.348 | 0.0124 |
| *AMIGO1* | -0.404 | 0.00425 |  | *MARK4* | -0.344 | 0.0140 |
| *CXCR4* | 0.405 | 0.00434 |  | *SFRP4* | 0.341 | 0.0155 |
| *PCOLCE* | 0.401 | 0.00477 |  | *RBBP7* | 0.337 | 0.0174 |
| *PHLDA2* | 0.398 | 0.00520 |  | *NR2F1* | 0.334 | 0.0178 |
| *INVS* | 0.395 | 0.00557 |  | *SOX9* | 0.328 | 0.0181 |
| *NRSN1* | -0.396 | 0.00557 |  | *C16orf80* | -0.335 | 0.0181 |
| *DLX5* | 0.395 | 0.00567 |  | *GTF2IRD1* | -0.334 | 0.0181 |
| *GSS* | 0.390 | 0.00647 |  | *NR5A2* | 0.329 | 0.0181 |
| *PROK1* | -0.387 | 0.00728 |  | *EIF2AK3* | 0.329 | 0.0181 |
| *SLIT1* | 0.385 | 0.00785 |  | *LEPR* | 0.330 | 0.0182 |
| *EXT2* | 0.383 | 0.00821 |  | *MBNL1* | 0.334 | 0.0185 |
| *HOXC4* | -0.382 | 0.00826 |  | *PRKAR1A* | 0.331 | 0.0185 |
| *FREM1* | -0.379 | 0.00902 |  | *CRMP1* | -0.331 | 0.0186 |
| *HHAT* | 0.376 | 0.00977 |  | *ACVR1B* | -0.332 | 0.0186 |
| *QKI* | 0.376 | 0.00990 |  | *ARHGAP24* | 0.330 | 0.0187 |
| *PLCE1* | 0.374 | 0.00998 |  | *NTRK2* | 0.325 | 0.0197 |
| *HES6* | 0.374 | 0.01001 |  | *MAEA* | -0.325 | 0.0199 |
| *CITED1* | 0.375 | 0.01010 |  | *DGCR2* | -0.320 | 0.0235 |
| *TSHZ2* | -0.372 | 0.01022 |  | *NCAM1* | -0.319 | 0.0236 |
| *KEAP1* | 0.373 | 0.01026 |  | *INA* | -0.317 | 0.0250 |
| *KIF1B* | -0.371 | 0.01035 |  | *TNFSF11* | 0.316 | 0.0254 |
| *SPEG* | -0.370 | 0.01058 |  | *PAFAH1B3* | -0.314 | 0.0260 |
| *RNF103* | -0.369 | 0.01075 |  | *BICC1* | 0.315 | 0.0262 |
| *NHLH2* | 0.368 | 0.01080 |  | *MEGF8* | -0.313 | 0.0267 |
| *CRABP1* | 0.367 | 0.01084 |  | *TFAP2B* | -0.311 | 0.0272 |
| *SIX2* | 0.368 | 0.01089 |  | *PDGFC* | 0.312 | 0.0273 |
| *KRT19* | -0.369 | 0.01093 |  | *DPYSL3* | -0.312 | 0.0274 |
| *TEAD4* | 0.367 | 0.01101 |  | *ARNT2* | -0.308 | 0.0300 |
| *FAM132B* | 0.366 | 0.01114 |  | *CXCL1* | 0.307 | 0.0301 |
| *EBF4* | -0.364 | 0.01124 |  | *SPEG* | -0.306 | 0.0308 |
| *TWIST1* | 0.364 | 0.01131 |  | *CRKL* | -0.305 | 0.0319 |
| *VCAN* | 0.365 | 0.01132 |  | *GATA2* | -0.304 | 0.0321 |
| *TMOD2* | -0.364 | 0.01135 |  | *AGT* | 0.301 | 0.0358 |
| *ITGA8* | -0.362 | 0.01165 |  | *HOXD4* | 0.297 | 0.0410 |
| *PCDHB12* | -0.362 | 0.01166 |  | *PLXNA1* | -0.296 | 0.0416 |
| *GRHL1* | -0.359 | 0.01168 |  | *AES* | -0.295 | 0.0419 |
| *LMO4* | 0.361 | 0.01180 |  | *FLT1* | 0.292 | 0.0454 |
| *APBA2* | -0.360 | 0.01181 |  | *BDNF* | 0.291 | 0.0470 |
| *NDRG4* | -0.359 | 0.01181 |  | *PCDHB11* | -0.291 | 0.0473 |
| *PAPSS1* | 0.359 | 0.01190 |  | *TNFRSF12A* | 0.288 | 0.0499 |
| *CSRP2* | 0.360 | 0.01193 |  | *BTG1* | 0.288 | 0.0501 |
| *TGFB2* | 0.359 | 0.01196 |  | *THBD* | 0.285 | 0.0548 |
| *FZD7* | 0.361 | 0.01198 |  | *HHEX* | 0.284 | 0.0551 |
| *SEMA3F* | 0.356 | 0.01244 |  | *FZD7* | 0.283 | 0.0573 |
| *SPATA18* | 0.356 | 0.01255 |  | *NEURL* | -0.282 | 0.0582 |
| *GDF11* | -0.356 | 0.01262 |  | *CALCA* | 0.280 | 0.0603 |
| *PCDHB15* | -0.353 | 0.01370 |  | *CLPTM1* | -0.280 | 0.0603 |
| *MIB1* | -0.350 | 0.01508 |  | *TGFB3* | 0.279 | 0.0617 |
| *MMP2* | 0.348 | 0.01611 |  | *SPG7* | -0.277 | 0.0623 |
| *FEZ1* | 0.347 | 0.01673 |  | *MATN3* | 0.278 | 0.0629 |
| *CHST9* | -0.346 | 0.01675 |  | *NTRK1* | -0.277 | 0.0629 |
| *HESX1* | 0.346 | 0.01686 |  | *PTEN* | 0.278 | 0.0632 |
| *MSH6* | 0.344 | 0.01764 |  | *PRMT1* | -0.275 | 0.0641 |
| *ZBTB16* | -0.343 | 0.01803 |  | *GRIK1* | 0.275 | 0.0645 |
| *ZMYM4* | -0.340 | 0.02018 |  | *QKI* | 0.274 | 0.0649 |
| *EBF1* | -0.339 | 0.02060 |  | *CTNNB1* | 0.275 | 0.0650 |
| *TLL2* | 0.337 | 0.02113 |  | *EYA2* | 0.273 | 0.0650 |
| *FKBP4* | 0.338 | 0.02121 |  | *DCN* | 0.275 | 0.0652 |
| *NEURL* | -0.337 | 0.02154 |  | *SMPD3* | -0.273 | 0.0655 |
| *TBX20* | -0.333 | 0.02430 |  | *HSD11B1* | 0.271 | 0.0690 |
| *PDGFRA* | 0.332 | 0.02441 |  | *PTMS* | -0.269 | 0.0732 |
| *NINJ1* | 0.331 | 0.02511 |  | *DOK4* | -0.268 | 0.0743 |
| *POU3F1* | -0.331 | 0.02519 |  | *GPR65* | 0.268 | 0.0748 |
| *DACT1* | 0.330 | 0.02579 |  | *SEMA3C* | 0.267 | 0.0752 |
| *WNT3* | 0.328 | 0.02682 |  | *FBN2* | 0.267 | 0.0754 |
| *CYFIP1* | 0.327 | 0.02779 |  | *SLC30A1* | 0.266 | 0.0754 |
| *SPHK1* | 0.327 | 0.02805 |  | *KEAP1* | -0.266 | 0.0758 |
| *PRRX2* | 0.324 | 0.02969 |  | *LAMA2* | 0.264 | 0.0783 |
| *SRF* | 0.324 | 0.02988 |  | *CA10* | -0.263 | 0.0812 |
| *CHODL* | 0.323 | 0.03039 |  | *SEMA5A* | 0.261 | 0.0813 |
| *FOXC1* | 0.322 | 0.03078 |  | *PHOX2B* | -0.261 | 0.0820 |
| *NRP1* | -0.322 | 0.03084 |  | *SVIL* | 0.262 | 0.0821 |
| *CTNND2* | -0.322 | 0.03085 |  | *CD164* | 0.261 | 0.0821 |
| *VANGL2* | -0.320 | 0.03210 |  | *NDRG4* | -0.262 | 0.0825 |
| *FZD2* | 0.320 | 0.03227 |  | *DIXDC1* | -0.261 | 0.0828 |
| *SHB* | 0.319 | 0.03269 |  | *EFNB3* | -0.261 | 0.0833 |
| *ALK* | 0.319 | 0.03309 |  | *FZD3* | -0.257 | 0.0842 |
| *DDX1* | 0.318 | 0.03363 |  | *FYN* | -0.259 | 0.0845 |
| *FGF14* | 0.317 | 0.03405 |  | *C4orf6* | 0.257 | 0.0850 |
| *HUS1* | 0.316 | 0.03429 |  | *CTNND2* | -0.257 | 0.0850 |
| *DRD2* | -0.316 | 0.03460 |  | *ITGB1* | 0.259 | 0.0850 |
| *GATA2* | -0.316 | 0.03471 |  | *ZFP36L1* | 0.256 | 0.0854 |
| *UGDH* | 0.315 | 0.03483 |  | *EDAR* | 0.257 | 0.0855 |
| *DNER* | -0.315 | 0.03499 |  | *TGFBR3* | 0.258 | 0.0861 |
| *ZNF22* | 0.315 | 0.03528 |  | *MAPT* | -0.258 | 0.0869 |
| *CEBPB* | 0.312 | 0.03729 |  | *FUT8* | 0.255 | 0.0870 |
| *ARVCF* | -0.310 | 0.04016 |  | *ARC* | 0.255 | 0.0883 |
| *ARHGAP22* | -0.309 | 0.04025 |  | *LMO1* | -0.254 | 0.0893 |
| *SOX4* | -0.309 | 0.04025 |  | *MT3* | 0.254 | 0.0895 |
| *SALL1* | -0.310 | 0.04043 |  | *VPRBP* | 0.253 | 0.0911 |
| *GRSF1* | 0.307 | 0.04287 |  | *AHNAK* | 0.252 | 0.0921 |
| *PHGDH* | 0.307 | 0.04293 |  | *GATA3* | -0.249 | 0.1008 |
| *HEXB* | 0.303 | 0.04704 |  | *PBX1* | -0.248 | 0.1017 |
| *DRP2* | 0.303 | 0.04765 |  | *GNAO1* | -0.247 | 0.1045 |
| *AQP4* | -0.301 | 0.04915 |  | *GSS* | 0.247 | 0.1045 |
| *SBF2* | -0.301 | 0.04929 |  | *RYK* | 0.247 | 0.1048 |
| *LIMD1* | 0.300 | 0.04944 |  | *WNT5A* | 0.246 | 0.1054 |
| *STRBP* | 0.300 | 0.04959 |  | *SCMH1* | -0.242 | 0.1065 |
| *HOXB4* | 0.300 | 0.04975 |  | *PLXNB2* | -0.245 | 0.1067 |
| *DSCAM* | 0.300 | 0.05010 |  | *SMAD1* | -0.242 | 0.1069 |
| *CNTN4* | 0.298 | 0.05228 |  | *TTPA* | 0.245 | 0.1070 |
| *BMPR2* | -0.298 | 0.05248 |  | *MCL1* | 0.242 | 0.1072 |
| *GJA1* | 0.297 | 0.05281 |  | *SEMA3E* | 0.242 | 0.1073 |
| *HOXA4* | 0.297 | 0.05304 |  | *TRO* | -0.244 | 0.1074 |
| *NCL* | 0.296 | 0.05311 |  | *THRA* | -0.244 | 0.1075 |
| *PRMT1* | 0.296 | 0.05342 |  | *GAS7* | 0.245 | 0.1075 |
| *TIMP1* | 0.296 | 0.05363 |  | *TNFRSF11B* | 0.245 | 0.1076 |
| *CNTFR* | -0.295 | 0.05511 |  | *PLXNB1* | -0.243 | 0.1077 |
| *AATF* | 0.294 | 0.05521 |  | *ARVCF* | -0.242 | 0.1079 |
| *MYCNOS* | 0.294 | 0.05527 |  | *PDPN* | 0.244 | 0.1080 |
| *DDX47* | 0.294 | 0.05534 |  | *CREG1* | 0.241 | 0.1085 |
| *PRELID1* | 0.293 | 0.05581 |  | *FGFR1* | 0.242 | 0.1086 |
| *CA10* | -0.290 | 0.06053 |  | *ACVR1* | 0.243 | 0.1087 |
| *PMP22* | -0.289 | 0.06185 |  | *PBX3* | -0.239 | 0.1093 |
| *LAMA3* | -0.289 | 0.06264 |  | *HOXB7* | 0.239 | 0.1100 |
| *EPHB1* | 0.287 | 0.06385 |  | *NFATC1* | 0.238 | 0.1100 |
| *SNTG2* | -0.287 | 0.06417 |  | *SEMA7A* | 0.240 | 0.1103 |
| *MACF1* | -0.287 | 0.06435 |  | *HTATIP2* | 0.238 | 0.1103 |
| *CAPN3* | -0.286 | 0.06543 |  | *SEMA3B* | 0.238 | 0.1104 |
| *FYN* | -0.286 | 0.06618 |  | *BMPR1B* | -0.240 | 0.1104 |
| *PTTG1IP* | 0.285 | 0.06731 |  | *MYL6B* | -0.240 | 0.1107 |
| *MYT1L* | -0.284 | 0.06814 |  | *SGCB* | -0.238 | 0.1111 |
| *PITX1* | 0.284 | 0.06880 |  | *DNM1L* | -0.239 | 0.1113 |
| *WWP1* | -0.283 | 0.07059 |  | *DCTN1* | -0.237 | 0.1118 |
| *WNT5B* | 0.282 | 0.07092 |  | *TP53BP2* | 0.237 | 0.1124 |
| *EXT1* | 0.280 | 0.07444 |  | *AHCTF1* | 0.236 | 0.1126 |
| *ADAMTS9* | 0.278 | 0.07654 |  | *LECT2* | 0.237 | 0.1127 |
| *APBB2* | 0.278 | 0.07687 |  | *HAND2* | -0.236 | 0.1128 |
| *SCG2* | -0.279 | 0.07700 |  | *NTNG1* | 0.236 | 0.1129 |
| *MTSS1* | -0.278 | 0.07730 |  | *PIAS4* | -0.236 | 0.1129 |
| *ACVR2A* | -0.278 | 0.07756 |  | *ERC1* | -0.235 | 0.1139 |
| *MYCN* | 0.277 | 0.07900 |  | *NPTX1* | 0.234 | 0.1151 |
| *EPHB2* | 0.276 | 0.07953 |  | *RNF103* | -0.233 | 0.1184 |
| *PNMA1* | 0.276 | 0.07987 |  | *NR4A3* | 0.233 | 0.1186 |
| *KIAA1715* | -0.275 | 0.08132 |  | *LGR4* | 0.233 | 0.1192 |
| *MYT1* | -0.275 | 0.08245 |  | *NDP* | 0.233 | 0.1196 |
| *BZW2* | 0.273 | 0.08489 |  | *DOC2A* | -0.231 | 0.1212 |
| *ASB1* | 0.273 | 0.08519 |  | *LHX3* | 0.231 | 0.1213 |
| *PCDHA3* | -0.273 | 0.08522 |  | *ADAM10* | 0.231 | 0.1215 |
| *TFAP2B* | -0.273 | 0.08538 |  | *PHC3* | 0.231 | 0.1215 |
| *NRGN* | 0.272 | 0.08649 |  | *CRIM1* | 0.231 | 0.1216 |
| *SIX4* | 0.271 | 0.08873 |  | *MPZ* | 0.229 | 0.1251 |
| *NAIP* | -0.270 | 0.08885 |  | *SEMA4C* | -0.229 | 0.1264 |
| *ELAVL3* | 0.270 | 0.09064 |  | *LGALS3* | 0.226 | 0.1285 |
| *DZIP1* | 0.269 | 0.09146 |  | *MKL2* | 0.226 | 0.1291 |
| *EDF1* | 0.268 | 0.09412 |  | *LECT1* | 0.227 | 0.1296 |
| *CBLN1* | 0.267 | 0.09544 |  | *SRI* | -0.228 | 0.1296 |
| *CALCRL* | -0.267 | 0.09580 |  | *TMOD2* | -0.227 | 0.1296 |
| *MST1R* | -0.266 | 0.09648 |  | *PCDHB6* | -0.227 | 0.1299 |
| *RACGAP1* | 0.265 | 0.09821 |  | *NAPA* | -0.227 | 0.1301 |
| *KALRN* | 0.265 | 0.09825 |  | *DHCR24* | 0.227 | 0.1305 |
| *MAEA* | 0.265 | 0.09849 |  | *EDA* | 0.225 | 0.1327 |
| *CHERP* | 0.265 | 0.09858 |  | *BIN1* | -0.224 | 0.1336 |
| *HOXD3* | 0.265 | 0.09867 |  | *TNFAIP1* | -0.225 | 0.1338 |
| *POMT1* | -0.264 | 0.09871 |  | *MAPK12* | -0.223 | 0.1360 |
| *ATR* | 0.263 | 0.10080 |  | *LIMD1* | 0.223 | 0.1363 |
| *WNT4* | -0.263 | 0.10086 |  | *BMP1* | -0.223 | 0.1365 |
| *PDPN* | 0.262 | 0.10108 |  | *MID1* | -0,223 | 0.1371 |
| *JPH1* | 0.263 | 0.10115 |  | *GJA1* | 0.222 | 0.1377 |
| *FES* | 0.263 | 0.10140 |  | *APLP1* | -0.222 | 0.1378 |
| *SEMA6C* | -0.262 | 0.10157 |  | *GHR* | 0.222 | 0.1384 |
| *GAL* | 0.261 | 0.10264 |  | *EMP1* | 0.222 | 0.1386 |
| *IL8* | 0.261 | 0.10355 |  | *ATP6AP1* | -0.221 | 0.1409 |
| *NMUR2* | -0.260 | 0.10363 |  | *CXCR4* | 0.220 | 0.1419 |
| *RORB* | -0.261 | 0.10364 |  | *SMAD3* | 0.220 | 0.1421 |
| *FMN2* | -0.260 | 0.10413 |  | *MTSS1* | -0.220 | 0.1421 |
| *ALDH3A2* | -0.259 | 0.10482 |  | *NRCAM* | -0.220 | 0.1422 |
| *SEMA3E* | -0.259 | 0.10533 |  | *AMOT* | 0.218 | 0.1451 |
| *HAND2* | -0.259 | 0.10580 |  | *PRKRA* | -0.218 | 0.1452 |
| *CANX* | 0.258 | 0.10648 |  | *NOTCH2* | 0.218 | 0.1454 |
| *TUBD1* | 0.258 | 0.10690 |  | *NES* | -0.218 | 0.1456 |
| *VAX2* | 0.258 | 0.10724 |  | *HMGCR* | 0.218 | 0.1457 |
| *TRIM54* | 0.257 | 0.10741 |  | *VLDLR* | 0.217 | 0.1479 |
| *NFE2* | -0.258 | 0.10785 |  | *RAI2* | 0.216 | 0.1480 |
| *TGFB3* | 0.257 | 0.10817 |  | *NRL* | 0.216 | 0.1483 |
| *MITF* | 0.257 | 0.10834 |  | *ANPEP* | 0.217 | 0.1487 |
| *SOX11* | 0.256 | 0.10958 |  | *ILK* | -0.216 | 0.1489 |
| *FGF11* | 0.256 | 0.10985 |  | *GATA6* | 0.216 | 0.1491 |
| *DLL3* | 0.255 | 0.11035 |  | *PTN* | 0.214 | 0.1527 |
| *CRYGD* | 0.255 | 0.11046 |  | *TAGLN3* | -0.215 | 0.1528 |
| *EIF2B5* | 0.255 | 0.11086 |  | *MYT1* | -0.214 | 0.1535 |
| *PSME4* | 0.255 | 0.11089 |  | *MAPK1* | -0.214 | 0.1541 |
| *CTNNBIP1* | -0.254 | 0.11102 |  | *CTNNBIP1* | -0.213 | 0.1567 |
| *EIF2AK3* | 0.255 | 0.11105 |  | *PTS* | -0.212 | 0.1596 |
| *ECE2* | -0.254 | 0.11151 |  | *EREG* | 0.212 | 0.1596 |
| *FGF2* | 0.254 | 0.11185 |  | *HAND1* | -0.211 | 0.1604 |
| *LAMA4* | -0.253 | 0.11206 |  | *GNRH1* | 0.210 | 0.1658 |
| *TP53* | 0.253 | 0.11258 |  | *YWHAH* | -0.210 | 0.1666 |
| *ITGA2* | 0.253 | 0.11401 |  | *RASA1* | 0.209 | 0.1681 |
| *GFRA3* | -0.252 | 0.11440 |  | *MDK* | -0.208 | 0.1697 |
| *SMAD3* | 0.252 | 0.11445 |  | *DRG1* | -0.207 | 0.1704 |
| *FZD5* | 0.251 | 0.11733 |  | *NEUROD1* | 0.207 | 0.1706 |
| *BMPR1A* | 0.250 | 0.11872 |  | *POGK* | -0.208 | 0.1706 |
| *UNC5D* | -0.250 | 0.11934 |  | *DMD* | 0.208 | 0.1708 |
| *ANGPTL4* | 0.250 | 0.11968 |  | *ZMYM4* | -0.208 | 0.1709 |
| *MEOX1* | -0.250 | 0.11983 |  | *DCLK1* | -0.207 | 0.1710 |
| *MCL1* | 0.249 | 0.11994 |  | *CACNA1A* | 0.206 | 0.1712 |
| *EGR3* | 0.249 | 0.12135 |  | *STX2* | -0.208 | 0.1713 |
| *E2F5* | 0.248 | 0.12247 |  | *F2* | 0.207 | 0.1713 |
| *DHCR7* | 0.248 | 0.12280 |  | *SEMA4F* | -0.206 | 0.1714 |
| *CNTN2* | 0.247 | 0.12411 |  | *BMP10* | 0.206 | 0.1716 |
| *SOX9* | 0.247 | 0.12478 |  | *TOP1* | 0.206 | 0.1718 |
| *CFC1* | 0.247 | 0.12531 |  | *NRP2* | -0.205 | 0.1725 |
| *SH3GL1* | 0.246 | 0.12547 |  | *BAX* | -0.205 | 0.1730 |
| *NEUROD1* | 0.246 | 0.12579 |  | *TRIM3* | -0.205 | 0.1733 |
| *STMN2* | -0.245 | 0.12747 |  | *NDUFV2* | -0.205 | 0.1737 |
| *FHL3* | 0.245 | 0.12760 |  | *FOXO4* | 0.204 | 0.1752 |
| *SMAD5* | -0.245 | 0.12762 |  | *ADAMTS9* | 0.204 | 0.1755 |
| *FLNB* | 0.244 | 0.13045 |  | *SEMA4D* | -0.204 | 0.1757 |
| *HECA* | -0.243 | 0.13439 |  | *IL8* | 0.204 | 0.1760 |
| *ACSBG1* | -0.242 | 0.13493 |  | *ROBO3* | -0.203 | 0.1765 |
| *HOXD9* | 0.242 | 0.13546 |  | *MYT1L* | -0.202 | 0.1788 |
| *EMD* | 0.242 | 0.13577 |  | *DKK1* | 0.202 | 0.1789 |
| *GLRB* | -0.241 | 0.13690 |  | *SPHK2* | -0.203 | 0.1791 |
| *HMGB3* | 0.241 | 0.13772 |  | *EGR2* | 0.201 | 0.1849 |
| *HTATIP2* | 0.240 | 0.14007 |  | *ANGPTL4* | 0.199 | 0.1926 |
| *ENC1* | 0.239 | 0.14218 |  | *WWP1* | 0.198 | 0.1942 |
| *DACH1* | 0.239 | 0.14220 |  | *FZD6* | 0.198 | 0.1952 |
| *DLK1* | 0.239 | 0.14341 |  | *PGF* | 0.198 | 0.1954 |
| *CLDN11* | 0.238 | 0.14426 |  | *RACGAP1* | 0.198 | 0.1957 |
| *ERCC2* | 0.238 | 0.14473 |  | *PTMA* | 0.197 | 0.1966 |
| *CENPF* | 0.237 | 0.14576 |  | *TMPRSS6* | 0.197 | 0.1972 |
| *PBX3* | 0.237 | 0.14641 |  | *TAL1* | 0.197 | 0.1977 |
| *CHRDL2* | 0.236 | 0.14816 |  | *DGAT1* | -0.196 | 0.1980 |
| *LAMA1* | 0.235 | 0.15238 |  | *PPAP2B* | 0.196 | 0.2009 |
| *IRS2* | 0.235 | 0.15245 |  | *PHLDA2* | 0.194 | 0.2022 |
| *ANGPT2* | 0.234 | 0.15342 |  | *OPHN1* | 0.193 | 0.2027 |
| *YWHAH* | -0.234 | 0.15371 |  | *MPPED2* | -0.194 | 0.2029 |
| *PKD2* | 0.234 | 0.15382 |  | *SOX2* | 0.194 | 0.2031 |
| *PTCH1* | 0.234 | 0.15387 |  | *DICER1* | 0.194 | 0.2033 |
| *ANGPTL2* | 0.234 | 0.15408 |  | *NF1* | -0.193 | 0.2034 |
| *GCNT2* | -0.234 | 0.15436 |  | *THBS1* | 0.194 | 0.2036 |
| *AXIN2* | 0.233 | 0.15505 |  | *PIK3CB* | 0.193 | 0.2037 |
| *MTL5* | -0.233 | 0.15528 |  | *EBF2* | 0.192 | 0.2037 |
| *BVES* | 0.233 | 0.15545 |  | *EXT1* | 0.192 | 0.2037 |
| *MAML3* | -0.233 | 0.15555 |  | *ZEB2* | 0.194 | 0.2039 |
| *FXR1* | 0.232 | 0.15627 |  | *MSX1* | 0.192 | 0.2039 |
| *RNH1* | 0.232 | 0.15674 |  | *OVOL2* | 0.194 | 0.2041 |
| *MARK4* | 0.231 | 0.15887 |  | *LUC7L* | -0.193 | 0.2042 |
| *COL11A2* | -0.231 | 0.15937 |  | *BMPR1A* | 0.192 | 0.2043 |
| *NDE1* | 0.231 | 0.15940 |  | *CASP7* | 0.193 | 0.2045 |
| *FUT10* | 0.231 | 0.15940 |  | *CDK5RAP1* | 0.194 | 0.2047 |
| *LOX* | 0.231 | 0.15948 |  | *NGRN* | -0.194 | 0.2054 |
| *MXD1* | -0.230 | 0.16084 |  | *TLX2* | -0.191 | 0.2058 |
| *CCNF* | 0.229 | 0.16404 |  | *DGKD* | -0.191 | 0.2061 |
| *ATRNL1* | -0.229 | 0.16436 |  | *MAP1S* | -0.194 | 0.2061 |
| *THRA* | -0.229 | 0.16460 |  | *FOXC1* | 0.191 | 0.2062 |
| *SIAH2* | 0.229 | 0.16487 |  | *ZNF3* | -0.191 | 0.2066 |
| *EHF* | -0.228 | 0.16603 |  | *ISL1* | -0.191 | 0.2069 |
| *LEFTY1* | -0.228 | 0.16658 |  | *EIF2B2* | 0.191 | 0.2070 |
| *WDR5* | 0.227 | 0.16701 |  | *SIX2* | 0.191 | 0.2071 |
| *THBD* | 0.227 | 0.16735 |  | *CDK5* | -0.190 | 0.2095 |
| *HRAS* | 0.227 | 0.16750 |  | *NGFR* | 0.189 | 0.2098 |
| *PSEN2* | 0.227 | 0.16769 |  | *SPP1* | 0.189 | 0.2102 |
| *PBX1* | -0.226 | 0.16781 |  | *NDRG2* | -0.189 | 0.2103 |
| *NHLH1* | 0.226 | 0.16781 |  | *MYCNOS* | 0.189 | 0.2104 |
| *AZU1* | -0.227 | 0.16786 |  | *ITGB7* | 0.189 | 0.2104 |
| *MYH3* | -0.225 | 0.17132 |  | *SPON1* | 0.189 | 0.2109 |
| *COL13A1* | 0.225 | 0.17218 |  | *ANGPTL3* | 0.188 | 0.2115 |
| *ZNF256* | 0.223 | 0.17691 |  | *GNRHR* | 0.187 | 0.2139 |
| *F2R* | 0.223 | 0.17706 |  | *JARID2* | -0.188 | 0.2142 |
| *HOXD10* | 0.223 | 0.17713 |  | *L1CAM* | -0.187 | 0.2146 |
| *EYA2* | 0.224 | 0.17722 |  | *NR4A2* | 0.187 | 0.2161 |
| *DIP2A* | -0.223 | 0.17789 |  | *ODAM* | 0.187 | 0.2163 |
| *DAZAP1* | 0.223 | 0.17796 |  | *OLIG2* | 0.186 | 0.2174 |
| *MNT* | 0.222 | 0.17908 |  | *GADD45B* | 0.186 | 0.2184 |
| *KLF5* | -0.222 | 0.17925 |  | *FZD1* | 0.185 | 0.2207 |
| *ZNF7* | 0.222 | 0.17966 |  | *SPHK1* | 0.185 | 0.2234 |
| *TCF12* | 0.221 | 0.18073 |  | *MMP19* | 0.184 | 0.2244 |
| *ETV5* | 0.221 | 0.18075 |  | *CDK5R1* | -0.184 | 0.2257 |
| *MAPT* | -0.221 | 0.18082 |  | *MMP11* | -0.184 | 0.2259 |
| *TIMM8A* | 0.221 | 0.18095 |  | *PHC1* | -0.182 | 0.2288 |
| *CHD5* | -0.221 | 0.18129 |  | *PIM1* | 0.183 | 0.2291 |
| *CHRD* | -0.221 | 0.18142 |  | *NHLH2* | 0.183 | 0.2299 |
| *ALDH5A1* | 0.220 | 0.18190 |  | *PAX2* | 0.182 | 0.2305 |
| *RYK* | 0.220 | 0.18253 |  | *MYL1* | 0.183 | 0.2306 |
| *ID2* | 0.220 | 0.18292 |  | *MEF2C* | 0.182 | 0.2321 |
| *PROK2* | 0.219 | 0.18437 |  | *ZBTB17* | -0.181 | 0.2322 |
| *CHKB* | -0.219 | 0.18504 |  | *WNT6* | 0.180 | 0.2369 |
| *COL4A4* | -0.218 | 0.18784 |  | *CRYGC* | -0.180 | 0.2375 |
| *ERBB3* | -0.218 | 0.18786 |  | *MITF* | 0.180 | 0.2375 |
| *WNT6* | 0.217 | 0.18948 |  | *REG3A* | 0.180 | 0.2377 |
| *ERBB4* | 0.218 | 0.18963 |  | *DPYSL4* | -0.179 | 0.2397 |
| *EIF2B4* | 0.217 | 0.19228 |  | *FHL1* | -0.179 | 0.2401 |
| *LAMB1* | 0.216 | 0.19279 |  | *RNH1* | -0.179 | 0.2402 |
| *LY6H* | 0.216 | 0.19313 |  | *RET* | 0.177 | 0.2460 |
| *EPO* | -0.216 | 0.19340 |  | *KDR* | 0.177 | 0.2464 |
| *TBX2* | 0.216 | 0.19380 |  | *ELAVL1* | 0.178 | 0.2465 |
| *C11orf73* | 0.215 | 0.19417 |  | *AGGF1* | -0.177 | 0.2468 |
| *DNASE2* | 0.215 | 0.19449 |  | *IL23A* | -0.177 | 0.2469 |
| *RELA* | 0.215 | 0.19504 |  | *GPM6B* | 0.177 | 0.2473 |
| *SMO* | 0.214 | 0.19955 |  | *STIL* | 0.177 | 0.2475 |
| *LMO1* | -0.214 | 0.19979 |  | *NEUROD4* | 0.177 | 0.2476 |
| *LETM1* | 0.213 | 0.19991 |  | *DAZAP1* | 0.176 | 0.2479 |
| *MAPK1* | -0.214 | 0.19991 |  | *DOPEY2* | -0.175 | 0.2517 |
| *FOXD3* | -0.213 | 0.20344 |  | *TRPS1* | 0.175 | 0.2523 |
| *GLDN* | 0.212 | 0.20548 |  | *XAB2* | -0.175 | 0.2529 |
| *AFF3* | -0.212 | 0.20557 |  | *HEXB* | 0.174 | 0.2539 |
| *IFRD1* | -0.212 | 0.20582 |  | *HPCAL4* | -0.175 | 0.2539 |
| *PAK3* | -0.212 | 0.20584 |  | *SRF* | 0.174 | 0.2563 |
| *PKP2* | -0.211 | 0.20686 |  | *SPRED2* | 0.173 | 0.2595 |
| *PTP4A1* | -0.211 | 0.20708 |  | *GNA13* | -0.172 | 0.2646 |
| *LGALS1* | 0.210 | 0.20986 |  | *HEY1* | 0.172 | 0.2647 |
| *CCM2* | -0.210 | 0.20995 |  | *APBB2* | 0.172 | 0.2648 |
| *SORT1* | 0.210 | 0.21009 |  | *DCX* | -0.171 | 0.2660 |
| *FZD6* | 0.210 | 0.21018 |  | *CHRDL1* | 0.171 | 0.2665 |
| *SH3GL2* | 0.209 | 0.21251 |  | *PDGFRA* | 0.171 | 0.2667 |
| *RPS6KA6* | 0.209 | 0.21293 |  | *PDE3B* | 0.171 | 0.2672 |
| *ARC* | 0.209 | 0.21310 |  | *CSPG5* | -0.169 | 0.2759 |
| *FGF1* | 0.208 | 0.21431 |  | *BMP2* | 0.169 | 0.2761 |
| *AES* | -0.208 | 0.21493 |  | *IRS2* | -0.169 | 0.2767 |
| *TRIM3* | -0.207 | 0.21732 |  | *TPP1* | 0.169 | 0.2775 |
| *PCDHA2* | -0.207 | 0.21740 |  | *SIAH2* | -0.168 | 0.2794 |
| *HTR2B* | 0.207 | 0.21764 |  | *LRCH4* | 0.168 | 0.2795 |
| *ZEB2* | -0.207 | 0.21784 |  | *ELK3* | 0.168 | 0.2810 |
| *HHIP* | 0.206 | 0.22235 |  | *ITGB4* | 0.167 | 0.2820 |
| *TRAF6* | 0.206 | 0.22283 |  | *PRPS1* | 0.166 | 0.2865 |
| *MYL4* | -0.205 | 0.22319 |  | *TFIP11* | -0.166 | 0.2868 |
| *ISL1* | -0.205 | 0.22348 |  | *SYK* | 0.166 | 0.2872 |
| *SPRY4* | 0.205 | 0.22373 |  | *AR* | 0.166 | 0.2875 |
| *WNT8A* | -0.205 | 0.22432 |  | *EBP* | 0.165 | 0.2877 |
| *HEMGN* | -0.205 | 0,22473 |  | *PDLIM5* | 0.165 | 0.2877 |
| *PCP4* | -0.204 | 0.22835 |  | *SPP2* | 0.166 | 0.2878 |
| *CREG1* | 0.203 | 0.22985 |  | *MLL* | -0.166 | 0.2879 |
| *MYH6* | -0.203 | 0.23100 |  | *MAP2K1* | -0.165 | 0.2880 |
| *SFRP4* | 0.203 | 0.23214 |  | *LMO4* | 0.165 | 0.2888 |
| *HOXA5* | 0.202 | 0.23392 |  | *HOXA7* | 0.164 | 0.2902 |
| *CALCA* | 0.202 | 0.23402 |  | *KIF1B* | -0.164 | 0.2911 |
| *CFDP1* | 0.201 | 0.23719 |  | *ANGPTL2* | 0.164 | 0.2918 |
| *CDON* | -0.201 | 0.23834 |  | *NRP1* | -0.164 | 0.2920 |
| *ELF3* | -0.200 | 0.24000 |  | *UNC5C* | -0.164 | 0.2920 |
| *VDR* | 0.200 | 0.24100 |  | *FGF12* | 0.163 | 0.2931 |
| *ID4* | 0.200 | 0.24133 |  | *RPS6KA3* | 0.163 | 0.2931 |
| *NR4A3* | 0.198 | 0.24182 |  | *MKKS* | -0.163 | 0.2933 |
| *GAP43* | -0.198 | 0.24220 |  | *LMO2* | 0.162 | 0.2936 |
| *E2F1* | 0.199 | 0.24239 |  | *PPT1* | 0.161 | 0.2936 |
| *HOXB8* | 0.199 | 0.24251 |  | *ERBB3* | 0.163 | 0.2937 |
| *MEA1* | 0.198 | 0.24276 |  | *UBE3A* | -0.162 | 0.2937 |
| *AMOT* | -0.199 | 0.24276 |  | *CHL1* | 0.163 | 0.2939 |
| *TMPRSS6* | -0.199 | 0.24283 |  | *CYLC1* | 0.162 | 0.2940 |
| *DIXDC1* | -0.199 | 0.24293 |  | *CYR61* | 0.162 | 0.2943 |
| *GREM1* | -0.199 | 0.24305 |  | *ENPEP* | 0.162 | 0.2945 |
| *DSCAML1* | 0.199 | 0.24309 |  | *CSRP2* | 0.162 | 0.2947 |
| *SPRED1* | 0.198 | 0.24326 |  | *JAG1* | 0.162 | 0.2948 |
| *BMP3* | -0.199 | 0.24358 |  | *FEZ1* | -0.159 | 0.2949 |
| *NLGN1* | 0.199 | 0.24397 |  | *SMAD4* | -0.159 | 0.2949 |
| *PAFAH1B3* | 0.197 | 0.24492 |  | *GPC3* | 0.162 | 0.2949 |
| *RAB23* | 0.197 | 0.24627 |  | *EMP2* | 0.159 | 0.2954 |
| *SEPP1* | -0.197 | 0.24637 |  | *FXR1* | 0.161 | 0.2955 |
| *IL1RAPL2* | -0.196 | 0.24655 |  | *VAMP5* | 0.161 | 0.2956 |
| *UNC5B* | 0.197 | 0.24671 |  | *FBN1* | 0.159 | 0.2957 |
| *LDB1* | 0.196 | 0.24698 |  | *SPRY1* | 0.159 | 0.2957 |
| *ACVR1B* | -0.196 | 0.24703 |  | *SEMA4G* | 0.161 | 0.2957 |
| *NPTX1* | 0.196 | 0.24768 |  | *LGI1* | 0.159 | 0.2957 |
| *ODAM* | 0.196 | 0.24803 |  | *MYH11* | 0.159 | 0.2959 |
| *TIMELESS* | 0.194 | 0.24989 |  | *MAFG* | -0.161 | 0.2963 |
| *VEGFC* | 0.194 | 0.25038 |  | *PLXNA3* | -0.159 | 0.2964 |
| *POSTN* | 0.194 | 0.25040 |  | *ENPP1* | 0.158 | 0.2969 |
| *PAX5* | 0.194 | 0.25075 |  | *JAG2* | -0.159 | 0.2971 |
| *EBP* | 0.194 | 0.25080 |  | *UTRN* | -0.158 | 0.2972 |
| *FOXO1* | -0.195 | 0.25095 |  | *MARK2* | -0.159 | 0.2975 |
| *NFATC3* | 0.194 | 0.25102 |  | *VCAN* | 0.160 | 0.2978 |
| *RPS4X* | 0.194 | 0.25125 |  | *ARHGAP22* | -0.160 | 0.2978 |
| *GLI2* | 0.195 | 0.25142 |  | *CYLC2* | 0.158 | 0.2982 |
| *FGFR3* | -0.195 | 0.25149 |  | *GAMT* | -0.160 | 0.2986 |
| *ZNF45* | 0.194 | 0.25159 |  | *SPIN1* | -0.160 | 0.2988 |
| *ADCYAP1R1* | 0.195 | 0.25164 |  | *ADAM22* | -0.160 | 0.2988 |
| *FGF13* | -0.195 | 0.25168 |  | *NBN* | 0.160 | 0.2990 |
| *GADD45G* | -0.193 | 0.25180 |  | *PITX1* | 0.157 | 0.2999 |
| *AVIL* | -0.193 | 0.25473 |  |  |  |  |
| *LGR4* | 0.192 | 0.25557 |  |  |  |  |
| *ROBO3* | -0.192 | 0.25594 |  |  |  |  |
| *NANOG* | -0.192 | 0.25829 |  |  |  |  |
| *ST6GAL2* | 0.191 | 0.26080 |  |  |  |  |
| *SVIL* | 0.191 | 0.26213 |  |  |  |  |
| *BDNF* | 0.190 | 0.26290 |  |  |  |  |
| *MTR* | 0.190 | 0.26325 |  |  |  |  |
| *ZEB1* | -0.190 | 0.26433 |  |  |  |  |
| *LDB2* | -0.190 | 0.26541 |  |  |  |  |
| *EFNB2* | -0.189 | 0.26886 |  |  |  |  |
| *SNAI1* | 0.188 | 0.27120 |  |  |  |  |
| *DOK4* | -0.188 | 0.27154 |  |  |  |  |
| *OBSCN* | -0.187 | 0.27476 |  |  |  |  |
| *ITGB8* | -0.188 | 0.27482 |  |  |  |  |
| *TOP2B* | 0.187 | 0.27491 |  |  |  |  |
| *PAFAH1B1* | -0.186 | 0.27868 |  |  |  |  |
| *DGKD* | -0.186 | 0.27880 |  |  |  |  |
| *DVL2* | 0.186 | 0.27905 |  |  |  |  |
| *HAND1* | -0.186 | 0.27917 |  |  |  |  |
| *GPR56* | 0.186 | 0.27919 |  |  |  |  |
| *EGR2* | 0.186 | 0.27944 |  |  |  |  |
| *ADAM18* | -0.186 | 0.27947 |  |  |  |  |
| *FGF18* | 0.186 | 0.27964 |  |  |  |  |
| *GNAO1* | -0.185 | 0.28210 |  |  |  |  |
| *SPG7* | -0.185 | 0.28298 |  |  |  |  |
| *CHRM1* | -0.185 | 0.28317 |  |  |  |  |
| *VGLL2* | -0.184 | 0.28560 |  |  |  |  |
| *CLC* | -0.184 | 0.28590 |  |  |  |  |
| *MKL2* | 0.184 | 0.28608 |  |  |  |  |
| *HLF* | -0.183 | 0.28769 |  |  |  |  |
| *STX2* | -0.183 | 0.28931 |  |  |  |  |
| *ATP2B2* | -0.182 | 0.29117 |  |  |  |  |
| *CEBPG* | 0.182 | 0.29260 |  |  |  |  |
| *ID3* | 0.181 | 0.29394 |  |  |  |  |
| *SEMA4C* | 0.182 | 0.29444 |  |  |  |  |
| *CYP46A1* | -0.181 | 0.29458 |  |  |  |  |
| *ETV4* | 0.181 | 0.29479 |  |  |  |  |
| *POU6F1* | -0.181 | 0.29506 |  |  |  |  |
| *PHF3* | -0.181 | 0.29570 |  |  |  |  |
| *HPCAL4* | -0.181 | 0.29581 |  |  |  |  |
| *BRSK2* | -0.180 | 0.29652 |  |  |  |  |
| *LGALS3* | 0.180 | 0.29692 |  |  |  |  |
| *PCDHA10* | -0.180 | 0.29782 |  |  |  |  |
| *AZI1* | 0.180 | 0.29806 |  |  |  |  |
| *EFNB1* | 0.180 | 0.29834 |  |  |  |  |
| *RPL29* | 0.180 | 0.29875 |  |  |  |  |
| *HOXC6* | -0.179 | 0.29904 |  |  |  |  |
| *LEFTY2* | -0.179 | 0.29926 |  |  |  |  |
